# Alkaline Phosphatase‐Activated NIR‐II AIEgens Nanosystem for Surgical and Postoperative Closed‐Loop Therapy of Advanced Osteosarcoma

**DOI:** 10.1002/advs.202516035

**Published:** 2026-01-20

**Authors:** Kaiyuan Liu, Ruotong Li, Li Zhang, Hengli Lu, Binhui Yang, Qian Hu, Yining Tao, Haoran Mu, Jing Han, Pengfei Zan, Jiakang Shen, Dongqing Zuo, Hongsheng Wang, Wei Sun, Xingjun Zhu

**Affiliations:** ^1^ School of Physical Science and Technology & State Key Laboratory of Advanced Medical Materials and Devices ShanghaiTech University Shanghai P. R. China; ^2^ Department of Bone Tumor Surgery Shanghai General Hospital Shanghai Jiao Tong University Shanghai P. R. China; ^3^ Department of Dermatology Shanghai Key Laboratory of Molecular Medical Mycology Shanghai Changzheng Hospital Naval Medical University Shanghai P. R. China; ^4^ Shanghai Clinical Research and Trial Center Shanghai China

**Keywords:** aggregation‐induced emission (AIE), alkaline phosphatase (ALP), immunotherapy, osteosarcoma, photothermal/photodynamic therapy (PTT/PDT)

## Abstract

In advanced osteosarcoma, tumor invasion often prevents complete resection, and immunotherapy is limited by the tumor's immunosuppressive nature, making residual lesions a key source of recurrence. To address this, we developed an ALP‐responsive theranostic nanoplatform (SGPF) integrating an AIEgens (STEA) and HSP90 inhibitor (Ganetespib) for imaging‐guided resection and multimodal therapy. Selenium‐doped STEA enables NIR‐IIb imaging and enhanced phototherapy via narrowed HOMO‐LUMO gaps and nonradiative decay optimization. At tumor sites, ALP‐triggered nanomicelle cleavage releases STEA and Ganetespib while vaporizing perfluorohexane to relieve hypoxia. NIR irradiation induces pyroptosis via caspase‐3/GSDME activation and immunogenic cell death, while Ganetespib suppresses glycolysis (HK2/PKM2 downregulation) to reverse lactate‐driven immunosuppression. This dual‐action strategy synergistically enhances T‐cell infiltration and ablates residual/metastatic lesions, offering a transformative approach for unresectable Osteosarcoma.

## Graphical abstract

This study designed a tumor microenvironment‐responsive AIEgen nanoparticle for near‐infrared photoimmunotherapy, which inhibits primary tumors and metastasis by promoting pyroptosis and suppressing aerobic glycolysis.

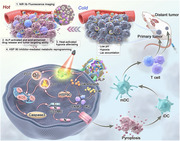

**Abbreviations**
OS: osteosarcoma
TME: tumor microenvironment
DAMP: danger associated molecular pattern
PTT and PDT: photothermal and photodynamic therapy
ICD: immunogenic cell death
AIE: Aggregation‐induced emission
ALP: Alkaline Phosphatase
ICT: intramolecular charge transfer
NMR: Nuclear Magnetic Resonance
TEM: transmission electron microscopy
DMSO: dimethyl sulfoxide
MD: Molecular dynamics
FBS: fetal bovine serum
DAPI: diamidino‐2‐phenylindole
ECAR: extracellular acidification rate
BMDC: bone marrow‐derived dendritic cell
CRT: Calreticulin
D–A–D: donor–acceptor–donor
SBTD: selenium‐substituted benzobisthiadiazole
AIE: aggregation‐induced emission
ISC: intersystem crossing
DLS: dynamic light scattering
EDS: energy‐dispersive X‐ray spectroscopy
MOMP: mitochondrial outer membrane permeabilization
LDH: lactate dehydrogenase
DC: dendritic cell; ECAR, extracellular acidification rate
OCR: oxygen consumption rate
MSEA: metabolite set enrichment analysis
dLN: draining lymph node
BMDC: bone marrow‐derived dendritic cell
CTL: cytotoxic T lymphocyte
H&E: hematoxylin and eosin
STEA: selenium‐substituted benzobisthiadiazole‐based tetraphenylethene‐triphenylamine AIEgen

## 1 Introduction

Osteosarcoma (OS) is a highly aggressive and heterogeneous primary malignant bone tumor. Despite recent advances in surgery, radiotherapy, chemotherapy, and targeted therapies, the 5‐year survival rate for patients has not been significantly improved [1, 2]. Particularly in advanced OS patients with tumors involving critical neurovascular structures, aggressive attempts at complete resection risk catastrophic intraoperative hemorrhage, mortality, or compromised limb function, thus precluding definitive tumor removal. Critically, residual lesion constitutes the primary risk factor for postoperative recurrence and metastasis, which adversely impacts survival outcomes [3, 4]. The emergence of tumor immunotherapy has offered new hope for advanced OS patients. However, a subset of patients exhibits poor responsiveness to immunotherapy, primarily attributed to the complexity of the tumor microenvironment (TME) and the development of treatment resistance [5, 6]. Among the diverse pathological mechanisms implicated, local tumor acidosis and hypoxia [7], infiltration of immunosuppressive cells [8], and tumor metabolic reprogramming [9] constitute critical factors contributing to suboptimal immunotherapy outcomes. There is an urgent need for alternative therapeutic strategies capable of multi‐target, multimodal synergistic action to overcome the current limitations of immunotherapy for advanced OS.

The TME of advanced OS is beset by interlocking pathological cues. Extensive bone mineralization compacts the extracellular matrix and constricts intratumoral vessels, throttling perfusion and engendering chronic hypoxia. Hypoxic stress activates HIF‐1α, which up‐regulates VEGF and PD‐L1, thereby compounding vascular dysfunction and deterring lymphocytic infiltration [10]. Concomitantly, a pronounced reliance on aerobic glycolysis floods the milieu with lactate, depressing the pH to below 6.5; this acidosis impairs dendritic‐cell maturation and favors the recruitment of M2‐polarized macrophages and regulatory T cells, amplifying immunosuppression. Although necrotic debris and bone fragments liberate danger‐associated molecular patterns (DAMPs) that could potentiate adaptive immunity, their antigenic potential is squandered: hyperactive HSP90 and a derailed antigen‐processing machinery preclude efficient cross‐presentation. Collectively, these structural, metabolic, and signaling aberrations cement OS as a quintessential “cold tumor,” largely unresponsive to contemporary immunotherapies. Notably, nanomaterial‐based photothermal and photodynamic therapy (PTT and PDT) can inflict direct cytotoxicity via local physicochemical reactions and induce immunogenic cell death (ICD) that liberates vast quantities of DAMPs and tumor‐associated antigens [11, 12]. This surge in antigen release markedly enhances antigen presentation, accelerates dendritic‐cell maturation, and fosters T‐cell infiltration, offering a distinctive avenue to convert a cold tumor into an immunologically “hot” one [13]. Yet amplified antigen exposure alone cannot overcome the lactate‐driven acidosis that prevails in the TME [14]. Hence, only by integrating PTT/PDT‐triggered immunogenic death with tumor re‐oxygenation, pH normalization, and glycolytic blockade can the multilayered immunosuppressive barriers be systematically dismantled and the microenvironment can be repolarized toward immune activation for the enhancement of therapeutic efficacy [15]. Current strategies, including antibody modification and ligand‐mediated targeting for improving treatment specificity, still face the challenges of target heterogeneity, nonspecific uptake, and inadequate drug exposure. In contrast, stimuli‐responsive nanomaterials rationally designed based on TME characteristics can be selectively activated at pathological sites, thereby increasing local drug accumulation and antigen exposure [16, 17]. With advantages such as structural simplicity, strong tissue‐penetration capability, and reduced systemic toxicity, these systems are particularly well‐suited for the treatment of advanced OS.

Aggregation‐induced emission luminogens (AIEgens) are regarded as ideal candidates for theranostic platforms because of their stable fluorescence in the aggregated state and their tunable energy‐dissipation pathways for phototherapy [18, 19, 20, 21]. The design of AIE molecules typically contains multiple freely rotating aromatic rotors that, in the molecularly dispersed state, dissipate excited‐state energy as heat through vigorous intramolecular motion [22, 23, 24]. Upon tight aggregation, the skeleton adopts a twisted conformation that suppresses undesirable intermolecular relaxation, thereby enhancing fluorescence intensity and promoting intersystem crossing [25, 26]. To red‐shift the second near‐infrared window (NIR‐II), most AIEgens are engineered either by extending the π‐conjugated backbone between donor and acceptor units or by introducing heavy atoms to narrow the HOMO–LUMO gap; both strategies concomitantly amplify PTT and PDT effects [27, 28]. Moreover, strengthening the intramolecular charge transfer (ICT) effect can promote excited‐state electron transfer for generating Type‐I ROS (including superoxide anion, O_2_^−^, and hydroxyl radical, ·OH) [29]. Unlike Type‐II ROS (singlet oxygen, ^1^O_2_), which relies on energy transfer, Type‐I ROS directly oxidizes biomolecules via electron transfer [30, 31]. Their generation is less sensitive to hypoxic microenvironments, and they possess a larger effective radius, enabling diffusion to distal cells to overcome limitations in photosensitizer distribution heterogeneity. Critically, they can synergize with tumor‐associated antigens (TAAs) released during pyroptosis to activate systemic immune responses.

**FIGURE 1:**
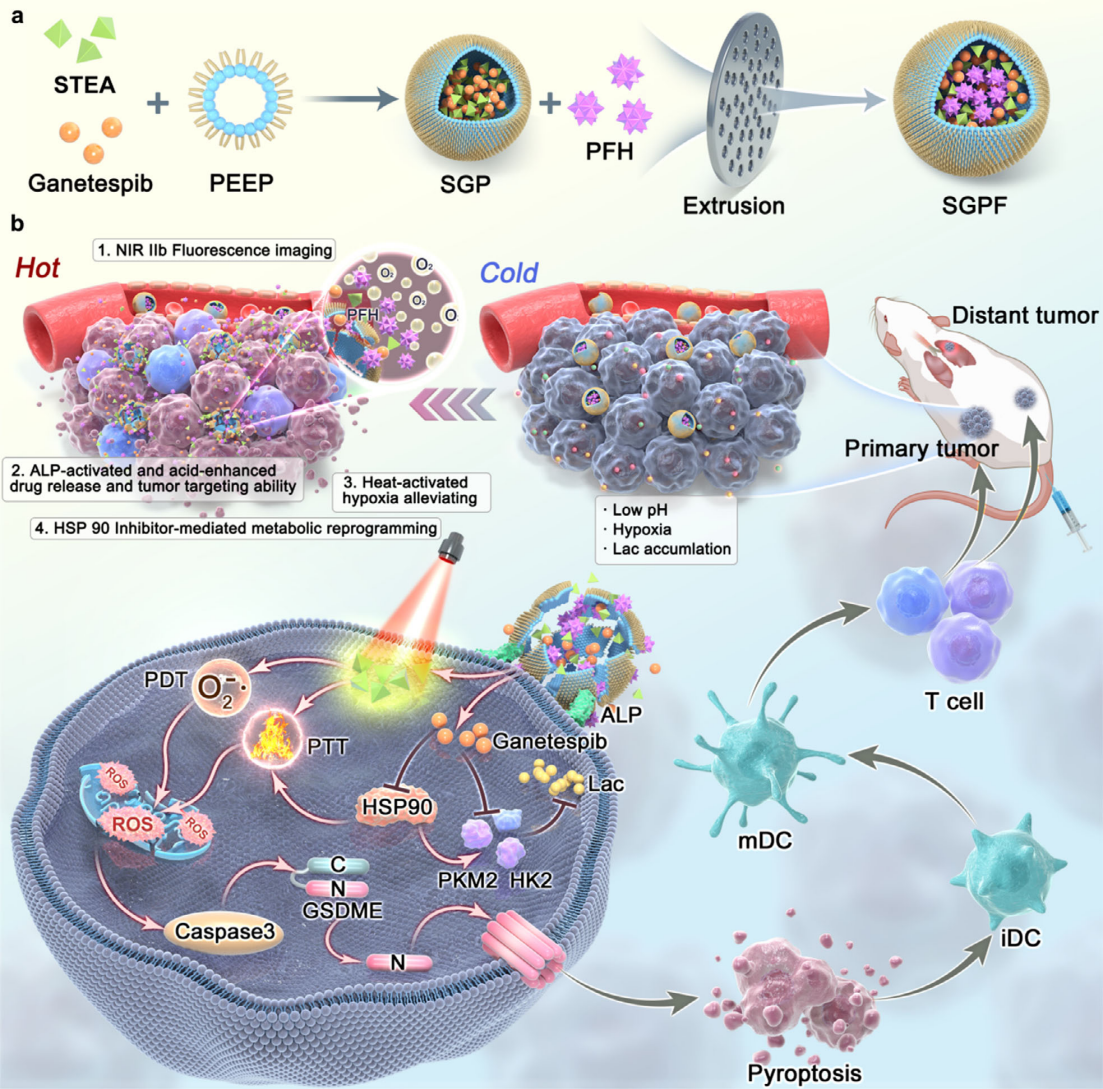
The schematic representation of the synthetic procedure and the mechanism underlying the antitumor and antimetastatic immune responses induced by the ALP‐activated NIR‐II nanoplatform. a) Diagrammatic depiction of the SGPF preparation process. b) Illustration of the decomposition procedure for SGPF nanoparticles in vivo and the synergistic photo‐triggered immunotherapy, which involves the inhibition of primary tumors and metastasis through the promotion of pyroptosis and the suppression of aerobic glycolysis.

Herein, an NIR‐II AIEgen‐based theranostic nanoplatform was established, where the AIE molecule STEA and the HSP90 inhibitor Ganetespib were co‐encapsulated within a polyphosphoester‐based micelle, followed by the loading of perfluorohexane (PFH) into the micellar core, forming the nanocomposite SGPF (Figure 1). Under 808 nm excitation, the fluorescence of STEA extends into the NIR‐IIb region, enabling precise tumor localization. Notably, the incorporation of selenium significantly narrows the HOMO–LUMO energy gap of STEA, endowing SGPF with excellent PTT and PDT therapeutic efficacy. Upon reaching tumor sites with high alkaline phosphatase (ALP) expression, SGPF undergoes gradual enzymatic degradation, leading to the release of payloads. The released STEA molecules can aggregate and generate enhanced phototherapeutic effect with 1064 nm light excitation that induces pyroptosis and ICD, and also vaporizes PFH, resulting in sustained oxygen release to relieve tumor hypoxia. In parallel, the released Ganetespib inhibits key enzymes involved in tumor glycolysis, thereby reducing local lactate accumulation. This immune‐favorable microenvironment promotes robust infiltration of immune effector cells, effectively converting the immunologically “cold” tumor into a “hot” one. The resulting systemic antitumor immune response also targets metastatic lesions, markedly reducing the incidence of distant metastases. Therefore, a “closed‐loop” theranostic strategy is proposed, in which intraoperative imaging–guided resection, immediate phototherapy for residual tumor ablation, and sustained postoperative immunotherapy via metabolic reprogramming are integrated. This self‐reinforcing framework provides a potential approach for modulating the tumor microenvironment toward an immunologically active state in advanced OS.

## 2 Materials and Methods

### 2.1 Materials

All chemicals for the synthesis were purchased from Sigma–Aldrich, Derthon Optoelectronic Materials Science Technology, and Titan Scientific as received without further purification. Phosphate‐buffered saline (1×PBS pH = 7.4), Dulbecco's modified Eagle's medium, and physiological saline were purchased from Sangon Biotech (Shanghai) Co., Ltd. 9,10‐anthracenediyl‐bis(methylene)dimalonic acid, Dihydrorhodamine 123, and Methylene blue were purchased from Sigma‐Aldrich.

### 2.2 Characterizations

Nuclear Magnetic Resonance (NMR) spectra were performed on a Bruker AVANCE NEO 400 spectrometer. Absorption curve was measured by Agilent Cary 5000 UV–vis–NIR spectrophotometer (Agilent Technologies). Mass Spectrometry (MS) was completed on Agilent 7890B‐7250 GC‐QTOF (USA). Fourier transform infrared (FTIR) was performed on A VERTEX 70 spectrometer. Transmission electron microscopy (TEM) images were carried out on a JEM‐2100 Plus emission transmission electron microscope operated at an acceleration voltage of 200 kV. HAADF‐STEM and elemental mapping images were tested by a JEM‐F200 transmission electron microscope. The near‐infrared emission spectra were obtained by using an NIR1700 spectrometer (Ideaoptics) irradiated by an 808 nm diode laser. Optical images were taken on a NIR‐II in vivo imaging system (Monet IGS‐1000, Suzhou NIR‐Optics Technologies Co., Ltd.)

### 2.3 Synthesis of SGPF

SGP was synthesized via a simple self‐assembly method. Specifically, 25 nmol of STEA, 8 mg of PEEP, and 0.3 mg of Ganetespib were dissolved in dichloromethane (DCM) and mixed thoroughly. The organic solvent was then removed using a rotary evaporator, allowing the dye and drug to co‐assemble with the polymer into a uniform thin film at the bottom of the flask. Subsequently, 0.4 mL of PBS was added, and the mixture was sonicated to disperse the micelles, thereby reducing size distribution variance. The final micelle solution was stored at 4°C, and the volume of PBS could be adjusted later according to experimental needs. SGPF was prepared based on the synthesized SGP. A defined volume of micelle solution was added to one side of a liposome extruder equipped with a 50 nm filter membrane, with the opposite side containing PFH. An equal volume of air was reserved in the syringe to ensure sufficient mixing of PFH with oxygen. The mixture was extruded back and forth 5–6 times to obtain SGPF. The resulting micelle solution was immediately stored at 4°C to prevent PFH evaporation.

### 2.4 AIE Performance Verification of STEA

STEA was dissolved in dimethyl sulfoxide (DMSO), and then DMSO and isopropanol solutions were mixed at designated volume ratios, ensuring that each solution contained the same molar amount of dye. The mixtures were sonicated for 10 min to obtain homogeneous solutions. The emission spectra of the materials under 808 nm excitation were measured using an NIR1700 spectrometer (Ideaoptics).

### 2.5 Photothermal Properties

200 µL of AIE NPs micelle solutions at different concentrations were added to a 96‐well plate. The 1064 nm laser was applied at a power density of 1 W cm^−^^2^, and a thermal imaging camera was used to monitor the real‐time temperature, with data recorded every 20 s until the temperature stabilized. For photothermal curves under different laser power densities, the concentration of AIE NPs was fixed at 15 µm, and the same procedure was followed until the temperature plateaued. In addition, the photothermal conversion efficiency of AIE NPs in aqueous solution was calculated according to the method reported in reference [32].

### 2.6 Photodynamic Performance

Singlet Oxygen (^1^O_2_): A solution of 9,10‐anthracenediyl‐bis(methylene)dimalonic acid (ABDA, 60 µM) dissolved in DMSO was mixed with SGPF and Ce6 solution (2 mL, 2 µm). The absorbance of the solution was recorded before irradiation. The mixture was then irradiated with a 1064 or 660 nm laser at a power density of 1 W cm^−^^2^, and absorption spectra were collected at different time intervals.

Superoxide Anion (O_2_^−^·): A solution of dihydrorhodamine 123 (DHR 123, 10 µM) in ethanol was mixed with 2 µm SGPF and Ce6 solution. Emission spectra from 510 to 700 nm were recorded under 490 nm excitation. The cuvette was then exposed to a 1064 or 660 nm laser at 1 W cm^−^^2^, and fluorescence spectra were collected at various time points.

Hydroxyl Radical (·OH): In 2 mL of 2 µm SGPF and Ce6 solution, 50 µL of 400 µm methylene blue (MB) was added, and the absorbance was recorded prior to irradiation. The sample was irradiated with a 1064 or 660 nm laser at the specified power, and absorption spectra were recorded at different time intervals.

### 2.7 Relative Quantum Yield

The QY was referenced against the second near‐infrared fluorescent probe IR26 (0.5%) [33]. Initially, IR26 was dissolved in 1,2‐dichloroethane (DCE) to prepare five samples with varying absorbances (ranging between 0.01 and 0.1). Subsequently, the samples were excited at 808 and a 900 nm long‐pass filter was employed to collect spectra ranging from 900 to 1600 nm. The fluorescence intensity curves for each sample were integrated, and the absorbance and integrated fluorescence values for the five samples were linearly fitted to obtain the reference slope. The same procedure was repeated for STEA to obtain the corresponding sample slopes.

The quantum yield calculation is based on the following formula:

$$QY_{sample}=QY_{ref}\times \frac{slope_{sample}}{slope_{ref}}\times {\left(\frac{n_{sample}}{n_{ref}}\right)}^{2}$$

Here, the reference refers to IR26. And n represents the refractive index of the solvent, with n_sample_ indicating the materials environment and n_ref_ indicating DCE.

### 2.8 Density Functional Theory

Molecular geometry and energy levels were optimized with Gaussian 09 [34]. Geometry optimization of STEA was performed at the B3LYP/6‐31G(d) level. Time‐dependent density functional theory (TD‐DFT) at the same theoretical level was then employed to calculate the singlet and triplet energy levels as well as the spin–orbit coupling (SOC) coefficients.

### 2.9 Molecular Dynamics (MD) Simulations

The partial charge of the STEA molecule was calculated using the Gaussian 16 code [35], and the 6–311g(d,p) basis functions were applied [36]. The generation amber force field (GAFF) [37, 38] was used to parametrize all atoms, such as the bond parameters, angle parameters, and dihedral angles, and so on. The self‐assembly behavior of the STEA molecule was simulated by molecular dynamics (MD) simulation. First, twenty STEA molecules were randomly inserted into a cube box with a side length of 8.0 nm, and then a 100 ns equilibrium simulation was performed under the NVT ensemble. Further, the balanced simulation box was filled with water molecules (15859), and then the 100 ns equilibrium simulation was performed under NPT ensemble. The dihedral angles of molecules within the aggregate were measured.

### 2.10 Drug Release

500 µL of SGPF micelle solution was added to a dialysis bag with a molecular weight cutoff of 500 Da. The dialysis bag was then placed in a 250 mL transparent beaker containing 200 mL of PBS as the external solution. The pH of the external PBS was adjusted according to experimental conditions and continuously monitored and maintained. The beaker was placed on a shaker for constant agitation. At predetermined time points, 100 µL of the external solution was withdrawn, and the absorbance at 300 nm was measured using a microplate reader. The release rate of Ganetespib was calculated based on the following formula.

$$\frac{The\;released\;molar\;amount\;of\;\mathrm{Ganetespib}}{The\;loading\;amount\;of\;\mathrm{Ganetespib}\;inside\;SGPF.}\times 100\%$$

### 2.11 Cell Culture, Cytotoxicity Assessment, and Live/Dead Cell Viability

All cell lines were cultured in high‐glucose DMEM supplemented with 10% fetal bovine serum (FBS), 1% penicillin, and 1% streptomycin. Cells were maintained at 37°C in a humidified atmosphere containing 5% CO_2_. For CCK‐8 viability assays, LM8 cells were seeded into 96‐well plates and allowed to adhere overnight. The culture medium was then replaced with 100 µL of fresh medium containing graded concentrations of AIE NPs or SGPF nanoparticles with or without laser irradiation (1064 nm NIR, 1.5 w/cm^2^, 10 min) after 4 h incubation. Following another 24 h incubation, the medium was removed and replaced with 100 µL fresh medium containing 10% CCK‐8 reagent. After 2 h incubation at 37°C, absorbance was measured at 450 nm using a microplate reader. Cell viability was calculated according to the following formula: ^*^Viability (%) = [(A_s__a__m__p__l__e_—A_s__t__e__ri__l__e_) / (A_u__n__t__r__e__a__t__e_d—A_s__t__e__ri__l__e_)] × 100*, where A_s__a__m__p__l__e_, A_u__n__t__r__e__a__t__e_d, and A_s__t__e__ri__l__e_ represent absorbance values of treated cells, untreated controls, and sterile medium blanks, respectively. Live/dead staining was performed by seeding LM8 cells in 6‐well plates overnight. After 24 h SGPF treatment with or without irradiation, cells were stained with calcein‐AM (2 µm) and propidium iodide (PI, 4.5 µm) for 30 min at 37°C. Fluorescence imaging was conducted using an inverted fluorescence microscope (λ_e__x_/_e__m_ = 490/515 nm for calcein‐AM; 535/617 nm for PI).

### 2.12 In Vitro Cell Detection of ROS and JC‐1 Experiments

Intracellular ROS levels were measured using the DCFH‐DA fluorescent probe and quantitatively analyzed with a confocal laser scanning microscope. The procedure was as follows: Mouse osteosarcoma LM8 cells were seeded into glass‐bottom culture dishes at a density of 1 × 10^5^ cells per well and incubated at 37°C for 24 h. Subsequently, DMEM medium containing AIE NPs or SGPF (0.05 µm) was added, and the cells were further incubated for 2 h with or without NIR irradiation. The cells were then washed three times with PBS and incubated with 10 µm DCFH‐DA solution at 37°C for 20 min. After washing three times, cell nuclei were stained with 4',6‐diamidino‐2‐phenylindole (DAPI), followed by another three washes. Cellular morphology and fluorescence were observed and recorded using a confocal microscope.

For mitochondrial membrane potential detection using JC‐1, AIE NPs, or SGPF (0.05 µm) were added to the culture medium, and cells were incubated for 2 h after either NIR irradiation (1064 nm 1.5 w/cm^2^, 10 min) or dark conditions. After washing with PBS, the medium was replaced with FBS‐free medium containing 10 µg/mL JC‐1 dye, and the cells were incubated at 37°C for 30 min. Finally, cellular morphology was observed and recorded using a fluorescence microscope.

### 2.13 Seahorse XF Glycolysis Stress Assay, Expression of Heat Shock Proteins and Metabolic Enzymes, and Production of Lactic Acid

For the Seahorse XF glycolysis stress assay, the extracellular acidification rate (ECAR) was measured using the Seahorse XF Glycolysis Stress Test Kit from Agilent (#103015–100; Agilent Technologies) and the XF24 Extracellular Flux Analyzer, following a previously described protocol. In brief, cells were seeded at a density of 5× 10^4^ cells per well in Seahorse XF24 V7 PS Cell Culture Microplates and cultured with or without SGPF (0.05 µm) overnight. ECAR was measured in XF base medium supplemented with 2 mM glutamine (pH 7.4) following the sequential injection of glucose (10 mM), oligomycin (1 µm), and 2‐DG (50 mM). The following calculations were performed: Glycolysis: Maximum rate measurement before oligomycin injection minus the last rate measurement before glucose injection, representing the basal glycolysis level. Glycolytic Capacity: Maximum rate measurement after oligomycin injection minus the last rate measurement before glucose injection, representing the maximum glycolysis level.

LM8 cells (2 × 10^5^ per well) were seeded into 6‐well plates and incubated at either 37°C or 43°C for 4 h. The other cells were incubated with 0.05 µm SGPF, either with or without NIR irradiation at 1.5 W/cm^2^, and further incubated for 4 h. After incubation, the culture medium was collected for the detection of extracellular lactate levels and lysine lactylation. The expression levels of HSP90 and metabolic enzymes were analyzed by Western blot (WB) using monoclonal antibodies from Abcam.

### 2.14 Determination of Dendritic Cell Maturation, Polarization of Macrophages and Cytokine Release In Vitro

Bone marrow‐derived dendritic cells (BMDCs) were isolated from 6‐week‐old Balb/c mice. In vitro DC stimulation experiments were conducted using a 24‐well Transwell system equipped with 0.4 µm polycarbonate porous membranes. A total of 1 × 10^4^ LM8 cells were seeded in the upper chambers. Once the cells reached approximately 80% confluence, AIE NPs or SGPF (0.05 µm) were added, followed by irradiation or non‐irradiation with a 1064 nm laser at a power density of 1.5 W/cm^2^ for 10 min. BMDCs or macrophages were seeded in the lower chambers at a density of 5 × 10^4^ cells per well, and the Transwell inserts were placed above them for co‐incubation. After 24 h, BMDCs or macrophages and their supernatants were collected.

BMDCs or macrophages were analyzed by flow cytometry. DCs were stained for 15 min with 5 µL of anti‐CD11c BB515 (eBioscience, 0.2 mg/mL), 5 µL of anti‐CD86 BV650 (eBioscience, 0.2 mg/mL), and 5 µL of anti‐CD80 PE (eBioscience, 0.2 mg/mL). And macrophages were stained with anti‐CD206 and anti‐CD86. After staining, cells were centrifuged at 120 × g for 5 min, resuspended, and subjected to flow cytometric analysis.

### 2.15 Transcriptome and Metabolome Detection

LM8 cells were incubated with SGPF at a concentration of 0.01 µm for 4 h under standard culture conditions. Following incubation, the cells were exposed to 1064 nm irradiation (1.5 W/cm^2^) for 10 min, then maintained at 37°C in a humidified incubator for an additional 2 h. Subsequently, both treated and control (untreated) cells were harvested for transcriptomic sequencing analysis. For metabolomic profiling, an identical treatment protocol was employed: LM8 cells were cultured with 0.01 µm SGPF for 4 h, irradiated with NIR light (1064 nm, 1.5 W/cm^2^, 10 min), and then incubated overnight at 37°C. Following incubation, both cells and corresponding culture supernatants were collected for comprehensive metabolomic analysis.

### 2.16 In Vivo Early Anti‐Tumor Immune Response

#### 2.16.1 Animal Model Establishment and Experimental Procedures

All animal experiments were conducted in accordance with protocols approved by the Animal Ethics Committee of Shanghai General Hospital (2023SQ102). Female C3H mice weighing 18–24 g were subcutaneously inoculated in the left flank with 3 × 10^6^ LM8 OS cells to establish tumor‐bearing models. When tumor volumes reached approximately 60 mm^3^, the mice were randomly allocated into four experimental groups: (1) 0.9% saline (100 µL) as vehicle control, (2) NIR irradiation alone, (3) SGPF (35 µm, 100 µL) treatment alone, and (4) combined SGPF (35 µm, 100 µL) with NIR irradiation (1064 nm, 10 min, 1.5 W/cm^2^). For groups receiving photothermal treatment (groups 2 and 4), 1064 nm laser irradiation (1.5 W/cm^2^, 10 min) was administered 24 h post‐intravenous injection. In the in vivo photothermal therapy experiments, the mice were anesthetized and secured on the imaging platform, and an infrared thermal camera was used to monitor the tumor temperature in real time during laser irradiation. The temperature was maintained within 42–45°C.

#### 2.16.2 Tissue Collection and Immunological Analysis

All animals were euthanized three days post‐treatment for comprehensive immunological evaluation. Lymph nodes and spleens were aseptically harvested for flow cytometric analysis to assess DC maturation status in lymph nodes and T cell distribution patterns in splenic tissues. Prior to euthanasia, blood samples were collected via cardiac puncture, and serum levels of interleukin‐6 (IL‐6), interferon‐γ (IFN‐γ), and tumor necrosis factor‐α (TNF‐α) were quantified using commercial ELISA kits according to manufacturer protocols.

#### 2.16.3 Histopathological Examination

Fresh tumor specimens were immediately fixed in 4% paraformaldehyde, embedded in paraffin, and sectioned for subsequent histological analyses. HE staining and Ki‐67 staining were used to evaluate the proliferative activity of tumors. Calreticulin (CRT) expression was visualized through specific staining of tumor sections. Apoptotic cells within tumor tissues were identified by TUNEL assay, while tumor‐infiltrating lymphocytes were characterized through dual immunofluorescence staining for CD4^+^ and CD8^+^ T cell markers to evaluate their spatial distribution within the tumor microenvironment. Before the mice were sacrificed, blood was collected from the eye sockets for blood routine and blood biochemical tests. After the mice were sacrificed, the heart, liver, spleen, lung, and kidney organs were collected for HE staining to evaluate biological safety.

### 2.17 In Vivo Distant Tumor Inhibition and Anti‐Lung Metastasis Studies

#### 2.17.1 Animal Models and Experimental Procedures

Primary tumor models were established by subcutaneous injection of 3 × 10^6^ LM8 cells into the right flank of C3H mice. To simulate metastatic disease, a secondary challenge of 1 × 10^6^ LM8 cells was administered to the left flank three days later. When the primary tumors reached approximately 60 mm^3^ in volume, the mice were randomly allocated into six experimental groups: (1) saline control, (2) anti‐PD‐1 antibody monotherapy, (3) SGPF alone, (4) SGPF + anti‐PD‐1 antibody, (5) SGPF with NIR irradiation, and (6) combination therapy with SGPF, anti‐PD‐1 antibody, and NIR.

#### 2.17.2 Treatment Protocols and Monitoring

For photothermal treatment groups, 1064 nm laser irradiation at 1.5 W/cm^2^ was applied for 10 min following 24 h of intravenous agent administration. SGPF was administered at a concentration of 35 µm in 100 µL volumes, while anti‐PD‐1 antibody blockade was achieved through intraperitoneal injection of 15 mg/kg every other day. Tumor volumes and body weights were monitored every three days, with tumor size calculated using the standard ellipsoid formula (volume = length × width^2^/2).

#### 2.17.3 Metastasis Assessment and Immunological Analysis

Following completion of the intervention protocol, an experimental lung metastasis model was established via tail vein injection of 2 × 10^5^ LM8 cells. Pulmonary metastasis was quantified 14 days later through high‐resolution CT imaging followed by histological examination of H&E‐stained lung sections. The simulated metastatic tumors from the left flank were excised for comprehensive immune profiling by flow cytometry, including quantification of CTLs, T‐regs, and polarization states of tumor‐associated macrophages (M1/M2 phenotypes).

### 2.18 Statistical Analysis

All experimental data were expressed as mean ± standard deviation. Normality and homogeneity of variance were assessed using the Shapiro–Wilk and Levene's tests, respectively. Statistical comparisons between two groups were conducted using Student's t‐test or the Mann–Whitney U test, as appropriate based on normality and variance homogeneity. Statistical comparisons between multiple experimental groups were performed using one‐way analysis of variance (ANOVA) implemented in SPSS statistical software (Version 25; IBM Corporation, Armonk, NY, USA). The threshold for statistical significance was established at *p* < 0.05, with progressively significant differences denoted as follows: ^*^ *p* < 0.05, ^**^ *p* < 0.01, and ^***^ *p* < 0.001.

## 3 Results and Discussion

### 3.1 Synthesis and Characterization

**FIGURE 2:**
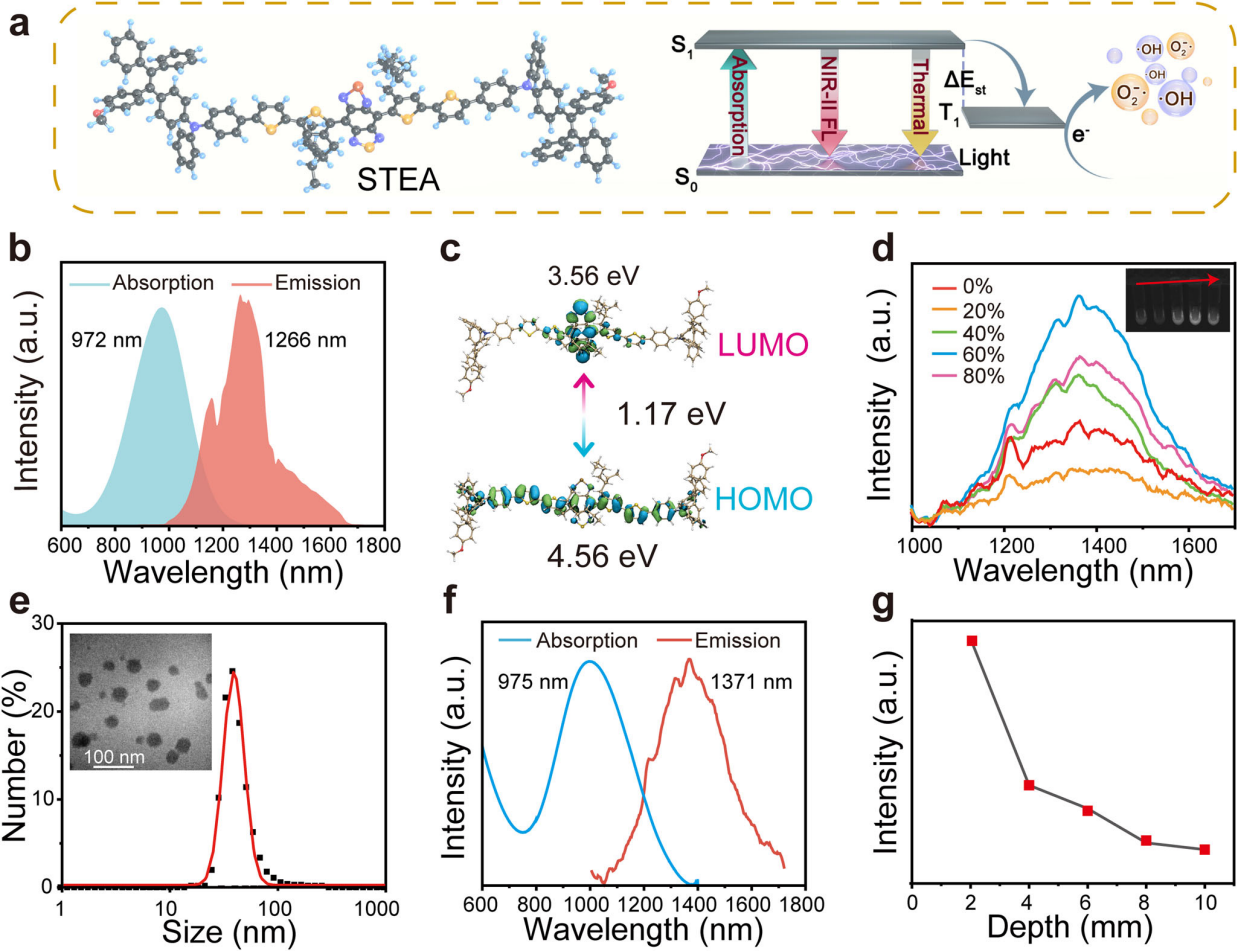
Structural and photophysical characterization of STEA and SGPF. (a) Molecular structure of STEA and schematic of photophysical process and ROS generation mechanism. (b) Absorption (blue) and NIR‐II fluorescence (red) spectra of STEA in aqueous solution. (c) HOMO and LUMO energy levels of STEA. (d) Fluorescence spectra of STEA in DMSO with varying isopropanol fractions (0–80%) (inset: corresponding imaging under 808 nm laser excitation). (e) Hydrodynamic diameter distribution of SGPF by DLS. Inset: TEM image (scale bar: 100 nm). (f) Absorption and emission profiles of SGPF in PBS (pH 7.4). (g) Quantified NIR‐II tissue penetration depth (0–10 mm) in chicken breast.

Extending the π‐conjugated framework on a strong donor–acceptor–donor (D–A–D) structure represents an effective strategy for developing fluorophores with long‐wavelength absorption and emission [24, 39]. However, the introduction of extended π‐bridges often increases molecular hydrophobicity, which predisposes the molecules to tight π–π stacking, thereby facilitating energy dissipation through nonradiative decay pathways. To address this, the dye molecule STEA was designed by covalently linking three key components: methoxy‐substituted tetraphenylethylene‐triphenylamine (TPE‐TPA) as peripheral donor units [22], sterically hindered bithiophene as the π‐bridge and primary electron donor, and a selenium‐substituted benzobisthiadiazole (SBTD) as the central strong electron acceptor (Figure 2a). The propeller‐like conformation of TPE‐TPA provides a twisted molecular geometry due to steric hindrance, imparting aggregation‐induced emission (AIE) characteristics. Additionally, its abundant free rotors enhance nonradiative thermal motion. The incorporation of heavy‐atom‐containing SBTD strengthens the D–A interaction, which not only reduces the energy gap but also facilitates the intersystem crossing (ISC) process [40]. The synthesis of STEA was confirmed by ^1^H, ^13^C NMR spectroscopy and mass spectrum (Figures S1–S3).

STEA exhibits a broad near‐infrared absorption profile ranging from 700 to 1200 nm, with a pronounced peak centered at 972 nm (Figure 2b). Notably, a substantial portion of the absorption resides within the NIR‐II window, enabling its applicability in subsequent phototherapy using NIR‐II laser irradiation. Upon excitation at 808 nm, STEA emits fluorescence with a maximum at 1266 nm and a relative quantum yield of 0.75%, with the emission extending up to 1700 nm within the NIR‐IIb region, which is advantageous for high‐resolution preoperative optical imaging. Further calculations revealed a narrow HOMO–LUMO energy gap of only 1.17 eV (Figure 2c). This reduced bandgap is primarily attributed to the strong electron‐withdrawing nature of the selenium‐containing acceptor, which also accounts for the red‐shifted absorption and emission characteristics of STEA. To investigate its AIE properties, STEA was subjected to solvent‐induced aggregation using mixtures of DMSO and isopropanol at varying ratios (Figure 2d). As the isopropanol fraction increased, the fluorescence intensity gradually enhanced, reaching its maximum at 60% isopropanol, indicating pronounced AIE behavior. A slight decrease in intensity at 80% isopropanol was observed, likely due to the precipitation of large STEA aggregates under poor solubility conditions.

ALP is a clinically recognized biomarker for the detection and monitoring of OS [41, 42], with expression levels in patients typically 100–317 times higher than those in normal tissues [43]. Compared to other common features of the tumor microenvironment, such as hypoxia, acidity, or glutamine dependence, ALP exhibits greater stability and clearer localization, being primarily distributed on the cell membrane and in the extracellular space. These characteristics make ALP a more suitable and accessible trigger for responsive drug delivery systems. Based on this reason, an amphiphilic triblock polymer with abundant phosphoester units, poly(ethyl ethylene phosphate) and poly(ϵ‐caprolactone) (PEEP‐PCL‐PEEP, hereafter referred to as PEEP, Figure S4), was synthesized and used to construct micelles, which could be degraded to release the payloads in tumor areas with high ALP activity [44, 45, 46]. Micelles were formed by encapsulating the dye STEA and the HSP90 inhibitor Ganetespib within the PEEP matrix, followed by repeated extrusion of PFH using a liposome extruder to load it into the micelles, generating a nanoplatform named SGPF. Transmission electron microscopy (TEM), dynamic light scattering (DLS), and energy‐dispersive X‐ray spectroscopy (EDS) confirmed the successful encapsulation of all three components (Figure 2e; Figure S5). Compared to the dye dissolved in solution, both the absorption and emission spectra of the micelles showed red‐shifted profiles, with the emission peak approaching 1400 nm (Figure 2f). Tissue penetration depth was simulated using chicken breast samples of varying thicknesses, and fluorescence intensity gradually decreased with increasing tissue thickness, with a detectable signal observed at depths up to 10 mm (Figure 2g).

### 3.2 PTT and PDT Evaluation

**FIGURE 3:**
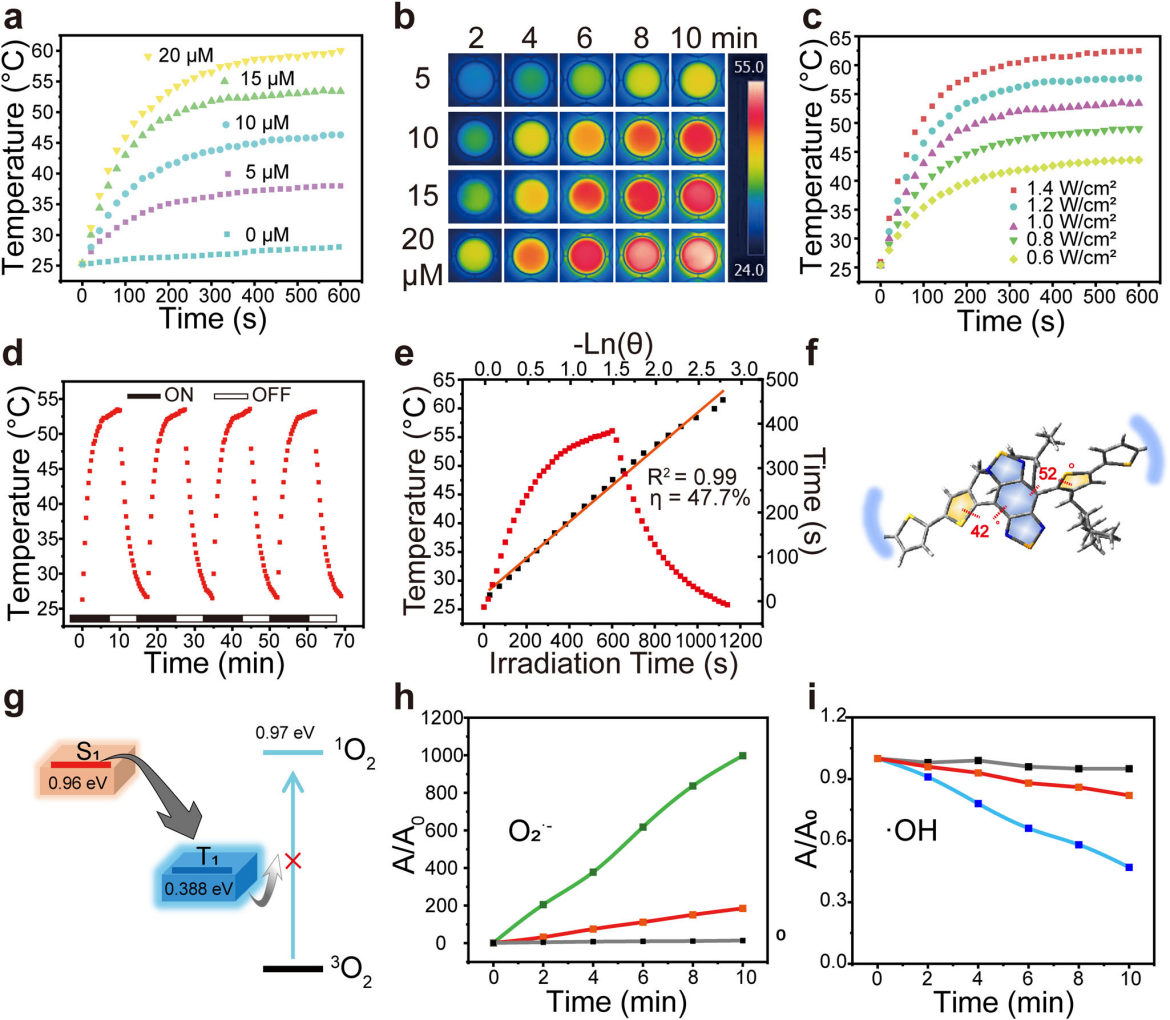
Photothermal and photodynamic characterization of SGPF nanoplatform. (a) Temperature elevation profiles of SGPF solutions (5–20 µm) under 1064 nm irradiation (1.0 W/cm^2^). (b) Infrared thermal images showing concentration‐ and time‐dependent heating. Color bar: 24–55°C. (c) Power density‐dependent heating kinetics of 15 µm SGPF (0.6–1.4 W/cm^2^). (d) Photothermal stability over four on/off cycles (1.0 W/cm^2^, 10 min ON/10 min OFF). (e) Photothermal conversion efficiency (η) calculation of SGPF. (f) Donor–acceptor dihedral angles of the STEA molecule, extracted from a representative conformation within the STEA aggregate during molecular dynamics simulation. (g) Triplet quenching mechanism for the production of ^1^O_2_. (h) Time‐dependent emission changes of DHR123, indicating ^•^O_2_^−^ generation. (i) Time‐dependent absorption changes of methylene blue indicating •OH generation. (The black line represents the group without SGPF, and the red line represents Ce6 under 660 nm irradiation (1 W/cm^2^)).

The photothermal conversion behavior of SGPF is illustrated in Figure 3a. Upon 1064 nm laser irradiation at a power density of 1 W cm^−^^2^, the temperature of the micelle solution rapidly increased within 1 min, primarily due to the twisted molecular conformation and the abundance of intramolecular rotors. After 5 min of irradiation, the temperature of micelles at all tested concentrations reached a steady state, with maximum temperatures of 53.5°C and 60°C observed for concentrations of 15 and 20 µm, respectively (Figure 3b). At a fixed concentration of 15 µm, temperature elevation was further evaluated under different power densities, and plateaus exceeding 62°C were achieved when the laser power was 1.4 W cm^−^^2^ (Figure 3c). A positive correlation was observed between the final temperature and either the concentration of the material or the laser power density. Notably, when the temperature exceeded 45°C, visible bubble formation was observed, indicating the gradual vaporization of PFH (Figures S6 and S7). In repeated heating–cooling cycles, the maximum temperature of SGPF remained nearly unchanged, confirming its excellent photothermal stability (Figure 3d). The photothermal conversion efficiency of SGPF was calculated to be 47.7%, which is higher than most previously reported NIR‐II photothermal agents (Figure 3e). Molecular dynamics simulations were employed to investigate the conformational changes of STEA upon aggregation in aqueous media. Within the aggregates, donor–acceptor dihedral angles of 42° and 52° were observed (Figure 3f), and intramolecular rotations facilitated the transition from a locally excited state to a twisted intramolecular charge transfer (TICT) state. Due to its sensitivity to various non‐radiative decay processes, the system exhibits greatly enhanced photothermal performance.

The photodynamic performance was subsequently evaluated under 1064 nm laser irradiation at a power density of 1 W cm^−^^2^. No significant degradation of ABDA was observed within 25 min of excitation, indicating negligible singlet oxygen generation (Figure S8). The calculated singlet (S_1_) and triplet (T_1_) state energies of STEA were 0.96 and 0.388 eV, respectively. Importantly, the T_1_ energy level (0.388 eV) was insufficient to sensitize ^3^O_2_ to ^1^O_2_ (0.97 eV), which accounts for the lack of singlet oxygen generation in many NIR‐II fluorophores (Figure 3g). The generation of superoxide anions (•O_2_^−^) and hydroxyl radicals (•OH) by SGPF was confirmed using dihydrorhodamine 123 (DHR123) and methylene blue as specific probes, indicating that the system possesses effective photodynamic activity (Figure 3h, i). Notably, when compared with the commercially available photosensitizer chlorin e6 (Ce6) under identical irradiation conditions, STEA exhibited superior ROS generation capability, further highlighting its advantage as an efficient photodynamic agent (Figure 3h,j). Similar observations have also been reported in other studies involving NIR‐II AIEgens [47], and the generation of superoxide anions can potentially contribute to subsequent immune activation.

### 3.3 ALP‐Responsive Degradation Enhances SGPF Functionality

**FIGURE 4:**
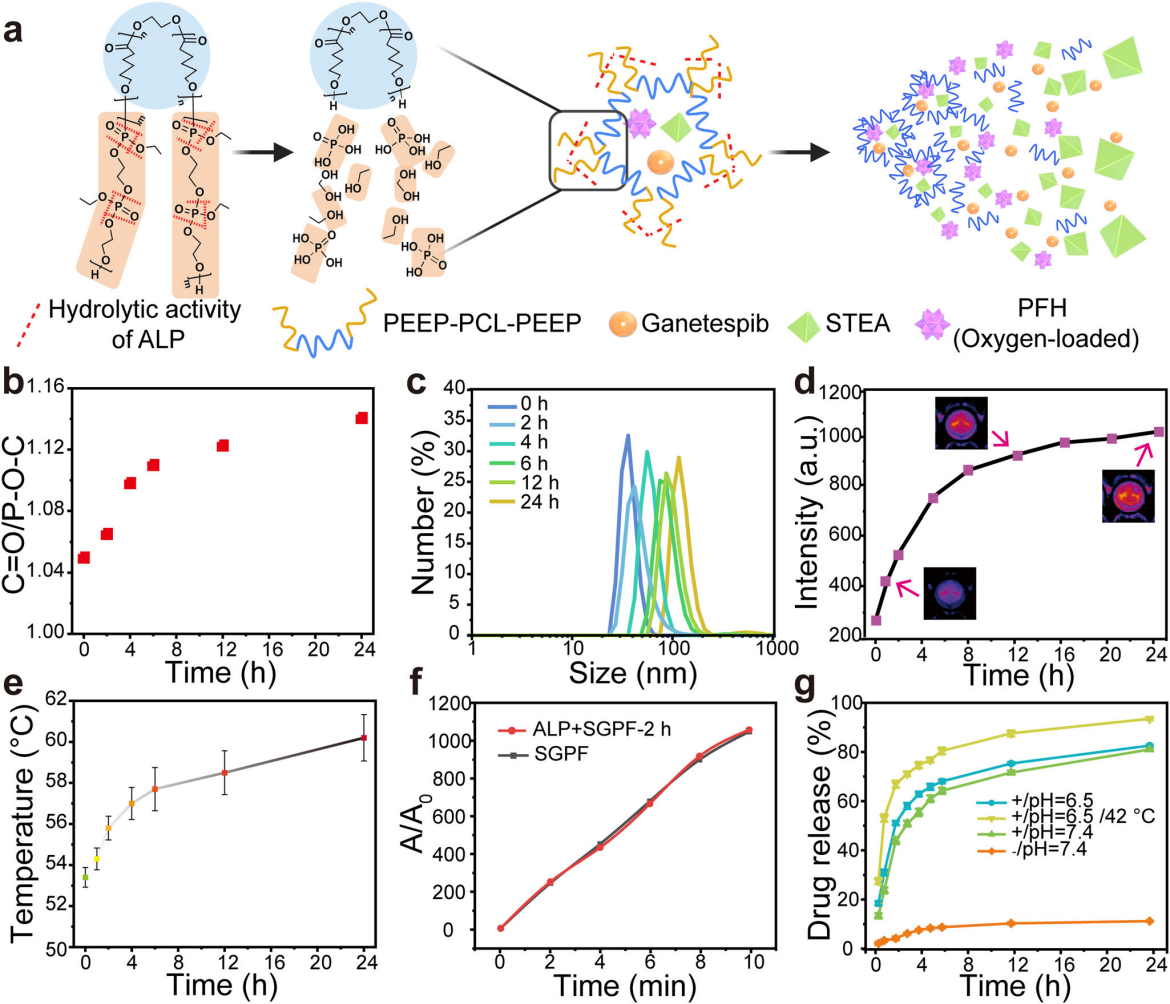
ALP‐responsive degradation enhances SGPF functionality. Activation of ALP leads to the degradation of SGPF and enhancement of its performance. (a) Schematic illustration of the ALP‐triggered hydrolysis of SGPF. (b) Time‐dependent FTIR analysis showing the ratio change of characteristic absorption peaks (C = O to P–O–C) to monitor the hydrolysis process. (c) Size distribution of SGPF measured at different time points after ALP treatment. (d) Fluorescence intensity changes of SGPF monitored over time with images as insets. (e) Temperature elevation profiles of SGPF at various ALP treatment durations (n = 3). (f) Comparison of ROS generation curves between untreated SGPF and ALP‐pretreated SGPF (2 h). (g) In vitro Ganetespib release profiles from SGPF under various pH and temperature conditions (n = 3).

The dynamic behavior of the micelles in response to ALP was systematically examined. Upon enzymatic stimulation, the phosphate ester bonds within the hydrophilic polymer chains were progressively cleaved, leading to the gradual exposure of the inner hydrophobic segments. These exposed hydrophobic components subsequently tended to self‐assemble into larger clusters under shear conditions mimicking blood flow (Figure 4a). This structural transition was substantiated by FTIR analysis, which tracked the changing ratio between ester groups in the hydrophobic core and phosphate bonds in the hydrophilic shell [32], confirming the stepwise cleavage of the hydrophilic domains (Figure 4b). DLS analysis revealed a continuous increase in micelle size over 24 h of ALP treatment. Specifically, the average particle size more than doubled at 6 h and exhibited a threefold increase after 24 h, relative to the initial state [48] (Figure 4c). But SGPF exhibited good stability in PBS and DMEM culture medium over a period of 7 days (Figure S9). This demonstrates the robust stability of the nanocomplex prior to reaching its target site, which is a prerequisite for effective systemic delivery and subsequent enzymatic activation. Simultaneously, an increase in oxygen evolution was monitored throughout the transformation process (Figure S10). This aggregation behavior aligns well with the AIE characteristics of the encapsulated molecules, as prolonged ALP exposure led to a steady enhancement in fluorescence intensity (Figure 4d), which is favorable for intraoperative fluorescence guidance during tumor resection. To maintain the balance between fluorescence and non‐radiative relaxation, different encapsulation ratios were evaluated. Although higher loading of STEA enhanced the photothermal and photodynamic effects, excessive loading caused a slight reduction in fluorescence intensity. Therefore, 25 nmol was selected to maintain sufficient fluorescence after enzyme‐induced aggregation while preserving therapeutic efficacy (Figure S11).

Concomitantly, changes in the photothermal and photodynamic effects of the micelles were assessed during degradation. A gradual rise in temperature was observed under laser irradiation following ALP incubation. After 6  h of enzymatic reaction, the temperature increased by 4.3°C, and further rose by 6.8°C at 24  h (Figure 4e). The photodynamic performance remained largely unchanged within the first 2  h of degradation (Figure 4f). Given that type I ROS generation predominantly depends on electron transfer rather than oxygen availability, the photodynamic effect can achieve broader cytotoxic coverage within hypoxic tumor microenvironments. In addition, the release behavior of Ganetespib from SGPF micelles was studied under varying conditions (Figure 4g), including ALP presence, pH, and temperature. Under neutral pH without enzymatic activity, the release was minimal, with only 11% released after 24  h. In contrast, ALP significantly accelerated the drug release, reaching 52% at 3  h and 83.5% at 24  h. The acidic environment (pH 6.5) mainly affects the early‐stage release behavior, leading to a moderately accelerated drug release in the early phase (e.g., within the first 6 h). As the release proceeds, ALP‐mediated cleavage of phosphate ester bonds becomes the dominant mechanism, resulting in comparable release profiles under different pH conditions at later time points. Furthermore, elevated temperature, which simulates the tumor microenvironment, markedly enhanced drug release. In the hyperthermia group, the release rate at 1  h was 1.3 times higher than that of the non‐heated control, and the cumulative release reached 96.6% at 24  h. Collectively, these results demonstrate that the ALP‐responsive micelle system not only enables spatiotemporal control over drug release but also offers synergistic advantages in photothermal‐assisted chemotherapy.

### 3.4 Cytotoxicity Assay for Therapeutic Efficiency of SGPF

**FIGURE 5:**
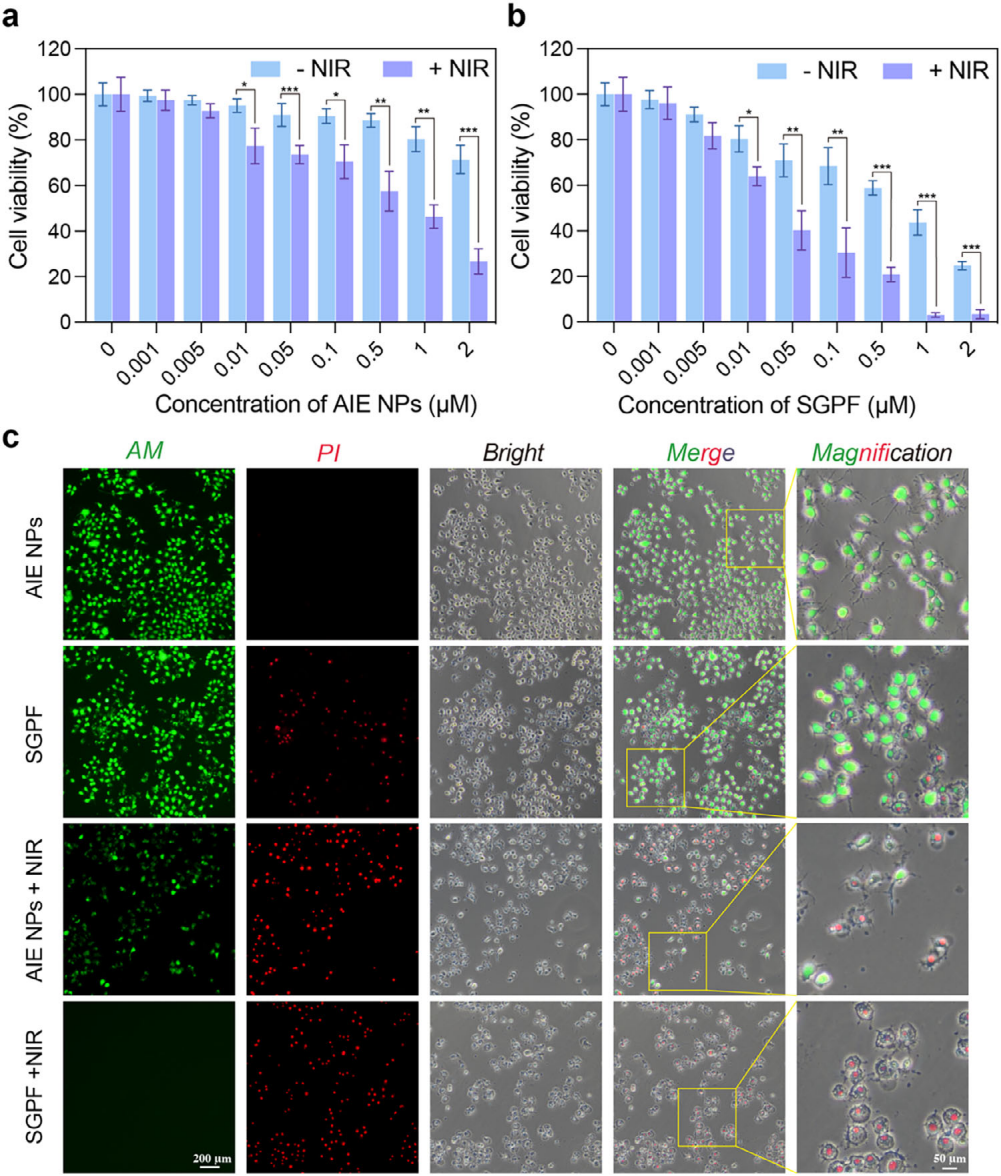
Cytotoxicity assessments of the AIE NPs and SGPF with or without NIR irradiation. (a, b) Viability of LM8 cells after 24 h incubation with indicated concentrations of AIE NPs or SGPF (0‐2 µm) ± NIR irradiation (1064 nm, 1.5w/cm^2^, 10 min) (n = 4). (c) Live/dead staining (Calcein‐AM/PI) of LM8 cells treated with 0.5 µm AIE NPs or SGPF ± NIR (1064 nm, 1.5w/cm^2^, 10 min). Scale bars: (c) 200 µM and 50 µm. Data are mean ± SD.Statistical significance: ^*^ *p* < 0.05, ^**^ *p* < 0.01, ^***^ *p* < 0.001 versus control group.

The SGPF nanoparticles demonstrated robust photothermal, photodynamic, and enzyme‐activated drug release capabilities in vitro. Subsequently, the tumor‐killing efficacy at the cellular level was systematically evaluated. To clarify the antitumor effect of the drug‐loaded micelles, the drug‐free micelles were designated as AIE NPs. The enzymatic responsiveness and selectivity of SGPF were further validated at the cellular level. Comparative studies using ALP‐high osteosarcoma cells (LM8) and ALP‐low control cells revealed a distinct difference in SGPF processing. As detailed in Figure S12, LM8 cells exhibited markedly brighter intracellular NIR fluorescence after co‐incubation with SGPF, compared to the minimal signal in ALP‐low cells. This contrast in AIEgen uptake and aggregation directly correlates with the differential ALP expression profile of the cells, providing strong evidence that the disassembly of SGPF and the release of its payload are selectively triggered by ALP activity. This result confirms the intended disease‐site specificity of our nanoplatform's activation mechanism. NIR confocal imaging revealed rapid cellular internalization of AIE molecules within 2 h of co‐culturing SGPF with OS cells (Figure S13). The cellular internalization of the SGPF nanoplatform is a sequential process initiated by extracellular ALP‐triggered degradation, as evidenced by the selective and time‐dependent increase in intracellular AIE fluorescence in ALP‐high LM8 cells compared to controls (Figures S10 and S11), which demonstrated the effective release and subsequent uptake of the payload. No significant effect on cellular viability was observed following NIR irradiation alone, as demonstrated in Figure S14. Further assessment of AIE NPs and SGPF on LM8 cells, with or without NIR irradiation, demonstrated that AIE NPs exhibited favorable biocompatibility with minimal cytotoxicity, while SGPF displayed potent tumor‐killing effects (IC_50_< 1 µm) (Figure 5a, b). This efficacy is mechanistically linked to HSP90 inhibitors. As a molecular chaperone, HSP90 stabilizes client proteins that drive tumor progression across malignancies, including myeloma and colon cancer [49, 50]. Prior studies confirm that HSP90 inhibition induces OS cell death through apoptosis promotion and cell cycle arrest [51]. Especially, 1064 nm NIR irradiation significantly enhanced tumor ablation when nanoparticle concentrations were over 0.01 µm, with efficacy exhibiting concentration‐dependent amplification‐consistent with PTT/PDT mechanisms. To further delineate the individual contributions of the therapeutic components within the SGPF nanoplatform, we performed additional in vitro cytotoxicity assays comparing the full formulation with nanoparticles selectively lacking either the Ganetespib or the PFH component, both with and without NIR irradiation (Figure S15). These experiments substantiate the distinct and complementary roles of each element in our combinatorial design. The results confirm that the phototherapeutic cell‐killing effect is intrinsically linked to the presence of the AIEgen, exhibiting a clear concentration‐dependent efficacy. Notably, nanoparticles formulated without PFH demonstrated a cytotoxicity profile nearly identical to that of the complete SGPF, underscoring the well‐documented biocompatibility and lack of inherent cytotoxicity of PFH [52]. This finding aligns with its primary, designed function as an inert oxygen carrier intended to alleviate tumor hypoxia and modulate the immune microenvironment rather than to exert direct cytotoxic action. Conversely, nanoparticles lacking Ganetespib but containing PFH showed significantly reduced cytotoxicity compared to SGPF, highlighting the HSP90 inhibitor as the principal source of the potent pharmacological cell‐killing effect. This is consistent with its established role as an anti‐tumor agent that disrupts oncogenic protein homeostasis [53]. In the context of our SGPF platform, Ganetespib's cytotoxicity is a crucial and intended facet of the therapeutic strategy, working in concert with phototherapy not only to eliminate tumor cells but also to promote a shift toward immunogenic cell death, thereby serving as a key driver for immune sensitization within the overall treatment paradigm.

PI/Calcein‐AM live‐dead staining was conducted to examine the extent of cell death induced by treatment (Figure 5c). While HSP90 inhibition alone triggered partial cell death, NIR irradiation substantially augmented lethality in SGPF‐treated cells. Bright‐field microscopy revealed pyroptotic morphology in LM8 cells under combined SGPF/NIR treatment: cellular swelling (ballooning) and membrane rupture—hallmarks of gasdermin‐mediated pyroptosis [54]. This aligns with reports that photothermal/dynamic therapies induce mitochondrial ROS‐mediated activation of gasdermin family proteins [55, 56]. Critically, pyroptosis represents a proinflammatory cell death modality distinct from apoptosis. Its induction in tumors may potentiate immune checkpoint inhibitors by remodeling the tumor microenvironment [57]. This mechanism not only addresses therapeutic resistance but also provides novel perspectives for immune activation and cancer vaccine development.

### 3.5 Synergistic Induction of Caspase3/GSDME‐mediated Pyroptosis by Phototherapy and SGPF In Vitro

**FIGURE 6:**
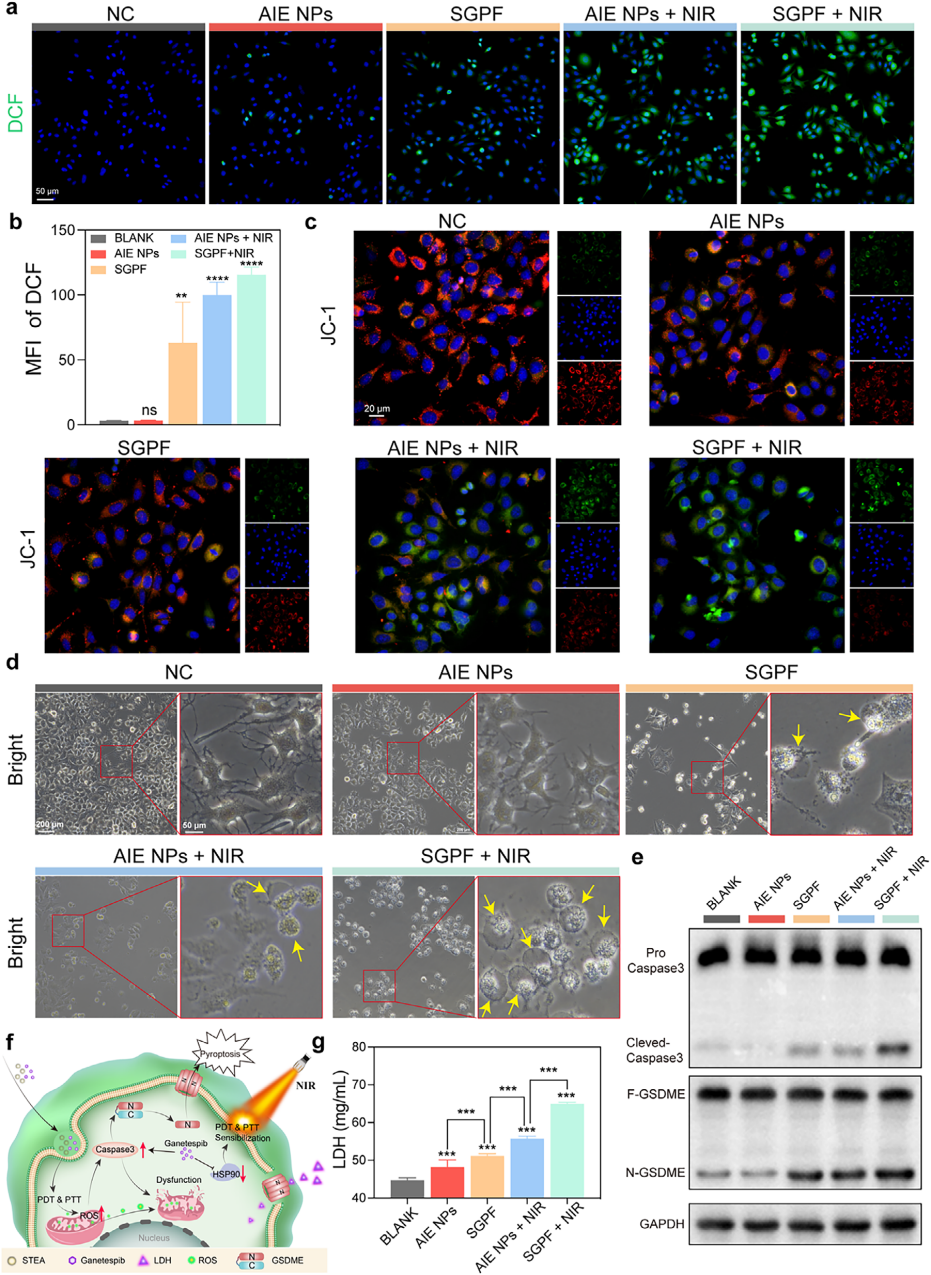
Synergistic induction of Caspase‐3/GSDME‐mediated pyroptosis by phototherapy and SGPF in vitro. (a) Intracellular ROS detection (DCFH‐DA fluorescence) in LM8 cells treated with PBS, AIE NPs, SGPF, AIE NPs + NIR, or SGPF + NIR. (b) Quantitative analysis of ROS fluorescence intensity from (a). Data represent mean ± SD (n = 3). (c) Mitochondrial membrane potential assessment (JC‐1 staining) in LM8 cells under the indicated treatments. Red: J‐aggregates (intact ΔΨm); Green: J‐monomers (depolarized ΔΨm). (d) Bright‐field images showing morphological changes in treated LM8 cells. (e) Western blot analysis of pyroptosis‐related proteins: GSDME (full‐length and N‐terminal fragment) and caspase‐3 (pro‐ and cleaved forms) in treated LM8 cells. GAPDH serves as a loading control. (f) The schematic diagram shows that ROS‐induced mitochondrial damage activates caspase‐3‐mediated GSDME cleavage, triggering pyroptosis. (g) LDH release quantification in cell culture supernatants post‐treatment (n = 4). Scale bars: (a) 50 µM; (c) 20 µM; (d) 200 and 50 µM. Data are mean ± SD. Statistical significance: ns indicated no significance, ^*^ *p* < 0.05, ^**^ *p* < 0.01, ^***^ *p* < 0.001 versus the control group.

The generation of elevated intracellular ROS levels resulting from •O_2_^−^ and •OH generation was quantified in LM8 cells using 2′,7′‐dichlorodihydrofluorescein diacetate (DCFH‐DA). Minimal ROS induction was observed upon SGPF treatment alone, whereas significant ROS amplification occurred following 1064 nm NIR irradiation, confirming effective photodynamic activation (Figure 6a, b). Prior evidence indicates both mild photothermia and HSP90 inhibition exacerbate mitochondrial damage, disrupting redox homeostasis [58, 59]. JC‐1 staining revealed substantial mitochondrial membrane potential (ΔΨ) dissipation in the SGPF+NIR group (Figure 6c). This electrochemical gradient (−150 to −180 mV), maintained by electron transport chain proton pumping, governs ATP synthesis and ROS regulation. Its collapse triggers mitochondrial outer membrane permeabilization (MOMP), facilitating cytochrome c release and caspase cascade initiation [60, 61]. Bright‐field microscopy (Figure 6d) identified heterogeneous cell death morphology in SGPF‐treated samples. Most cells exhibited classical apoptotic features, including cellular shrinkage, cytoplasmic condensation, and organelle compaction, while a minority displayed pyroptotic characteristics. NIR irradiation substantially increased pyroptotic cell prevalence, with SGPF+NIR showing significantly higher pyroptosis incidence than AIE NPs+NIR. Western blot analysis (Figure 6e) confirmed caspase‐3 activation and gasdermin E (GSDME) cleavage in SGPF‐mediated phototherapy, evidenced by accumulated N‐terminal GSDME fragments that execute pyroptotic pore formation. Although HSP90 inhibitors typically promote apoptosis, our phototherapeutic system redirected cell death toward pyroptosis (Figure 6f). This shift is mechanistically attributable to caspase pathway convergence: HSP90 inhibition amplifies caspase activity that, under dominant PDT/PTT‐induced pyroptotic signaling, preferentially cleaves GSDME rather than activating apoptotic effectors. In summary, we propose that the shift toward pyroptosis is driven by the synergistic creation of a high‐signal, pro‐inflammatory cellular context. Intense phototherapy‐generated ROS causes profound mitochondrial damage, triggering a robust caspase‐3 activation that exceeds the threshold for routine apoptosis. Meanwhile, HSP90 inhibition likely plays a permissive role by modulating the cellular state, potentially affecting the stability of death substrates. Within this specific milieu—characterized by elevated caspase‐3 activity and inflammatory cues—GSDME is efficiently cleaved. The resulting N‐terminal fragments oligomerize to form plasma membrane pores, thereby executing the observed immunogenic pyroptotic outcome. As shown in Figure 6g, lactate dehydrogenase (LDH) release assays provided functional validation of pyroptosis, as plasma membrane rupture during gasdermin‐mediated pore formation permits cytoplasmic LDH efflux. Pyroptosis represents an immunogenic cell death modality characterized by the release of TAAs and DAMPs. While pyroptosis induction enhances hyperthermia‐induced cytotoxicity, its capacity to potentiate immunotherapy efficacy remains constrained by immunosuppressive barriers within the TME. Consequently, further validation is warranted to determine whether SGPF nanoparticles can overcome these microenvironmental constraints through TME remodeling, thereby activating systemic antitumor immunity.

### 3.6 In Vitro Modulation of Immune Activation Accompanied by Targeting Aerobic Glycolysis in Tumor Cells

**FIGURE 7:**
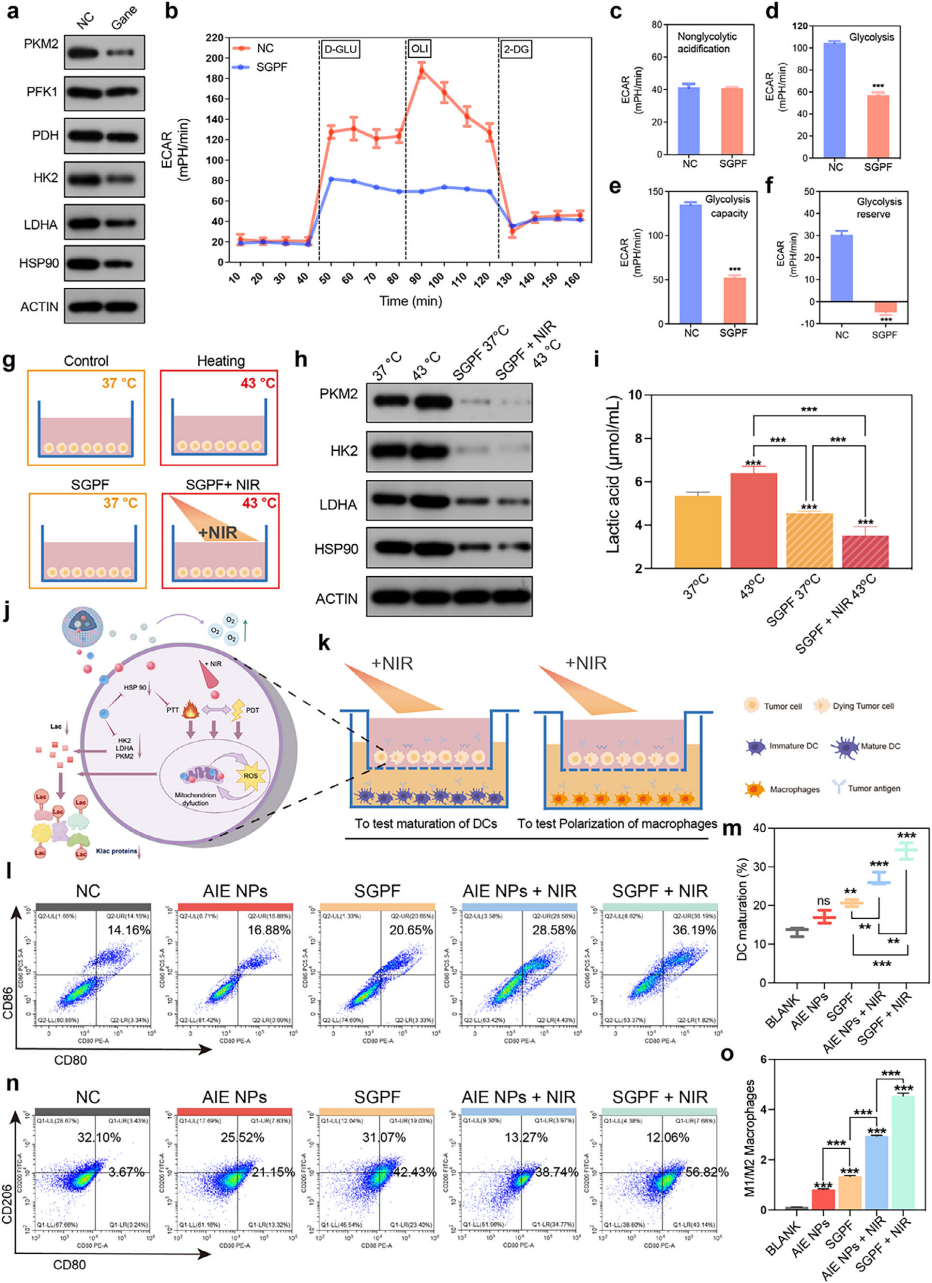
In vitro modulation of immune activation accompanied by targeting aerobic glycolysis in tumor cells. (a) Protein levels of glycolysis‐related enzymes (HK2, PKM2, LDHA) in LM8 cells treated with or without ganetespib (0.1 µm, 24 h). (b) Extracellular acidification rate (ECAR) profiles of LM8 cells treated with SGPF or control, measured using a Seahorse XFe96 Analyzer (n = 5). (c–f) Quantitative analysis of Seahorse XFe96‐derived glycolytic parameters: (c) non‐glycolytic acidification, (d) glycolysis, (e) glycolytic capacity, and (f) glycolytic reserve in LM8 cells upon SGPF treatment (n = 5). (g) Schematic of experimental design: LM8 cells cultured at 37°C or 43°C, treated with or without SGPF. (h) Protein levels of glycolysis‐related enzymes (HK2, PKM2, LDHA) and HSP90 in LM8 cells under the treatments described in (g). (i) Lactate concentration in the supernatant of LM8 cell cultures under the treatments described in (g) (n = 3). (j) Schematic illustration depicting SGPF‐mediated remodeling of the immunosuppressive tumor microenvironment through enhanced PTT/PDT efficacy and inhibition of key glycolytic enzymes to reduce lactate production. (k) Schematic of the co‐culture system: LM8 cells (upper chamber) treated with AIE NPs or SGPF ± 1064 nm NIR irradiation, co‐cultured with bone marrow‐derived dendritic cells (BMDCs) or macrophages (lower chamber). (l, m) Flow cytometry analysis (l) and quantification (m) of mature dendritic cells (CD86^+^CD80^+^ gated on CD11c^+^ cells) 24 h post‐treatment with PBS, AIE NPs, SGPF, AIE NPs + NIR, or SGPF + NIR in the co‐culture system (n = 5). (n, o) Flow cytometry analysis (n) and quantification (o) of macrophage polarization (CD206^+^ M2‐like and CD80^+^ M1‐like markers) 24 h post‐treatment with PBS, AIE NPs, SGPF, AIE NPs + NIR, or SGPF + NIR in the co‐culture system (n = 5). Data are mean ± SD. ns indicated no significance, ^*^ *p* < 0.05, ^**^ *p* < 0.01, ^***^ *p* < 0.001 versus control group.

Accumulation of lactate within the TME, driven by aerobic glycolysis, potently facilitates tumor growth and metastasis [62]. This metabolic rewiring provides energy and biosynthetic precursors to fuel proliferation while establishing an acidic milieu (pH <6.5) that induces epithelial‐mesenchymal transition and angiogenesis, thereby enhancing invasiveness. Concurrently, lactate remodels the TME into an immunosuppressive niche by inhibiting CD8^+^ T/NK cell cytotoxicity and IFN‐γ secretion [63], impairing dendritic cell (DC) maturation [64], and expanding immunosuppressive populations (Tregs, M2 macrophages, MDSCs). Such metabolic reprogramming directly compromises immune checkpoint blockade efficacy through T cell exhaustion and lactate‐induced lysine lactylation (e.g., MRE11 K673 modification) [65], which enhances DNA repair and confers therapeutic resistance. Chen et al. initially demonstrated that HSP90 inhibition via ganetespib suppresses glycolysis by dual‐targeting PKM2/PFKP, thereby alleviating IL‐8–mediated immunosuppression and enhancing radiotherapy efficacy in head and neck cancer [49]. In the current study, Western blot analysis confirmed that ganetespib similarly downregulates PKM2, HK2, and LDHA expression in OS (Figure 7a). Seahorse metabolic assays further revealed that SGPF nanoparticles (ganetespib‐loaded) significantly suppressed aerobic glycolysis in LM8 cells (Figure 7b). Concomitant reductions in extracellular acidification rate (ECAR) and oxygen consumption rate (OCR) indicated profound impairment of mitochondrial function and energy metabolism (Figure 7c–f). Notably, mild photothermal effect (40‐43°C), distinct from ablative hyperthermia, accelerates tumor metabolism and paradoxically exacerbates lactate production. When LM8 cells were subjected to 43°C, elevated expression of glycolytic rate‐limiting enzymes (PKM2, HK2, LDHA) and HSP90 was observed (Figure 7g). However, SGPF treatment counteracted this thermal adaptation, suppressing both HSP90 and metabolic enzymes even under hyperthermic conditions (Figure 7h). Critically, SGPF combined with NIR irradiation (achieving 43°C) synergistically reduced lactate accumulation in the supernatant (Figure 7i), indicating effective metabolic reprogramming. Western blot analysis of pan‐lysine lactylation was carried out in LM8 cells maintained at 37°C or 43°C, and treated at 37°C with SGPF, AIE NPs plus NIR, or SGPF plus NIR. Increased pan‐lysine lactylation of intracellular proteins was observed at elevated temperatures (43°C vs. 37°C), whereas this modification was effectively reversed by SGPF+NIR co‐treatment through attenuation of lactate accumulation (Figure S16). Previous studies have established that lactate accumulation‐induced lysine lactylation across multiple proteins promotes malignant phenotypes in tumors, including accelerated progression and immunotherapy resistance [66].

To assess the immunological consequences of lactate reduction, a transwell co‐culture system was established (Figure 7j, k). SGPF‐treated tumor cells promoted maturation of immature DCs, with enhanced efficacy upon NIR irradiation (Figure 7l, m). This effect is attributed to three complementary mechanisms: (i) PDT/PTT‐mediated immunogenic cell death releasing tumor antigens; (ii) pyroptosis‐triggered activation of antigen‐presenting cells; and (iii) lactate depletion alleviating DC functional suppression. Similarly, SGPF+NIR treatment polarized macrophages toward an antitumoral phenotype, as evidenced by decreased M2 markers (Figure 7n, o). Given that M2 macrophages secrete IL‐10/TGF‐β and express PD‐L1 to inhibit CD8^+^ T cells and recruit Tregs, their repolarization signifies TME remodeling. Collectively, SGPF‐mediated phototherapy achieves metabolic reprogramming that suppresses lactate secretion, synergizes with pyroptosis‐induced immunogenicity, and promotes DC maturation while inhibiting M2 macrophage polarization. This multifaceted strategy establishes a foundation for augmenting ICB efficacy. Previous studies have demonstrated that the inhibition of glycolysis can potentiate the anti‐tumor activity of immune cells. For instance, in triple‐negative breast cancer, limiting glycolysis was shown to suppress the expression of tumor‐derived cytokines such as G‐CSF and GM‐CSF, thereby reducing the accumulation of myeloid‐derived suppressor cells (MDSCs), enhancing T‐cell immunity, and ultimately restraining tumor growth and metastasis [67]. Furthermore, glycolysis inhibition can enhance immune cell function by attenuating lactate production. It has been reported that the suppression of glycolysis augments T cell‐mediated anti‐tumor immunity, demonstrating enhanced anti‐tumor efficacy in both in vitro and in vivo settings [68]. While our current study does not provide direct experimental proof that the observed immune activation is exclusively caused by glycolysis inhibition, the established literature strongly supports the role of targeting aerobic glycolysis in reversing the immunosuppressive tumor microenvironment. It should be noted that other factors induced by our combined treatment may also contribute synergistically to the overall immune activation.

**FIGURE 8:**
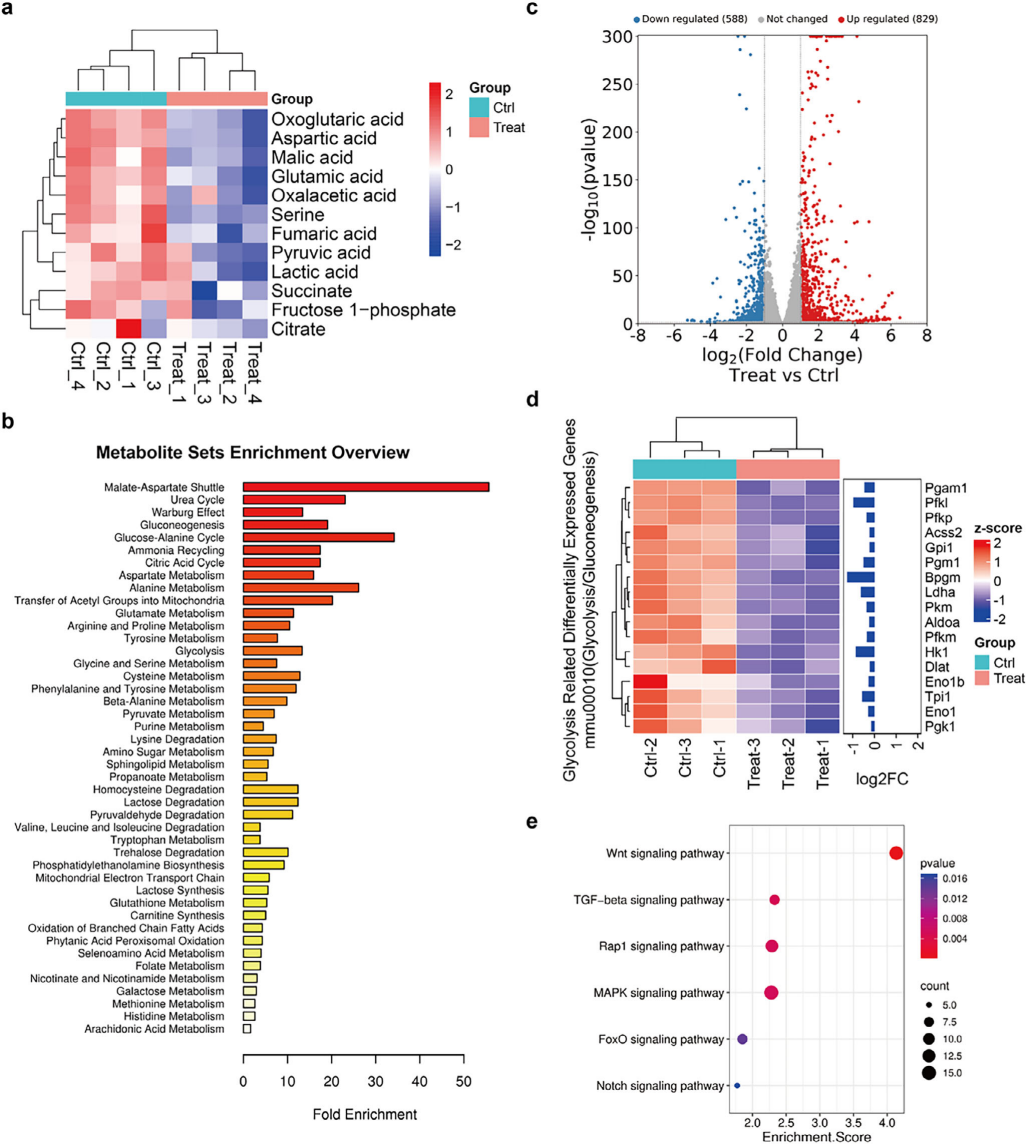
Metabolomic and transcriptomic profiling of SGPF plus NIR treatment. (a) Down‐regulated metabolite distribution in glycolytic pathways post‐treatment (n = 4). (b) Metabolite set enrichment analysis (MSEA) comparing experimental groups. (c) Volcano plot illustrating differentially expressed genes between treatment and control groups. (d) Transcriptional regulation of glycolysis‐associated genes revealed by RNA sequencing (n = 3). (e) Signaling pathway enrichment analysis of down‐regulated genes.

The combined therapeutic regimen of SGPF with near‐infrared (NIR) irradiation elicited a profound reprogramming of glycolytic metabolism, as evidenced by distinct alterations in key pathway intermediates (Figure 8a, b). Comparative analysis revealed significant downregulation of multiple glycolytic metabolites in the treatment group relative to controls, with lactate demonstrating particularly marked suppression. Notably, phosphoenolpyruvate and pyruvate levels were substantially reduced, suggesting either diminished metabolic flux through the terminal stages of glycolysis or enhanced diversion of pyruvate toward auxiliary biosynthetic pathways. These observations were further corroborated by metabolite set enrichment analysis (MSEA), which identified glycolysis as exhibiting notably high fold enrichment between experimental groups, collectively indicating a substantial perturbation in glycolytic flux and potential metabolic rewiring induced by the treatment.

Transcriptomic profiling yielded complementary insights, with volcano plot analysis (Figure 8c) revealing widespread differential gene expression between experimental and control groups. Subsequent examination of glycolysis‐associated genes (Figure 8d) demonstrated congruent transcriptional changes, though with an intriguing dissociation between mRNA and protein expression patterns. While no significant upregulation was observed for HK2 and PKM2 transcripts, their protein levels were markedly diminished—a phenomenon likely attributable to the HSP90 inhibitor's predominant role in modulating the post‐translational stability of these critical glycolytic enzymes rather than influencing their transcriptional regulation. Conversely, PKM1 expression was found to be downregulated, potentially contributing to the observed reduction in downstream metabolites, including pyruvate. These findings collectively suggest that the SGPF plus NIR therapeutic strategy may exert its suppressive effects on tumor cell aerobic glycolysis through both canonical and non‐canonical regulatory mechanisms.

Pathway enrichment analysis (Figure 8e) identified several signaling cascades with particularly high enrichment scores, including the Wnt, Notch, and MAPK pathways—all of which have been extensively documented in prior studies as critical regulators of glycolytic activation and tumor cell metabolic reprogramming. The coordinated modulation of these pathways provides compelling evidence that the SGPF plus NIR intervention elicits a multifaceted suppression of malignant phenotypes in tumor cells, simultaneously targeting their proliferative capacity and metabolic adaptability. These comprehensive findings establish a robust mechanistic foundation to support subsequent in vivo investigations and potential clinical translation of this innovative therapeutic approach.

### 3.7 Fluorescence Imaging and Early Immune Activation In Vivo

**FIGURE 9:**
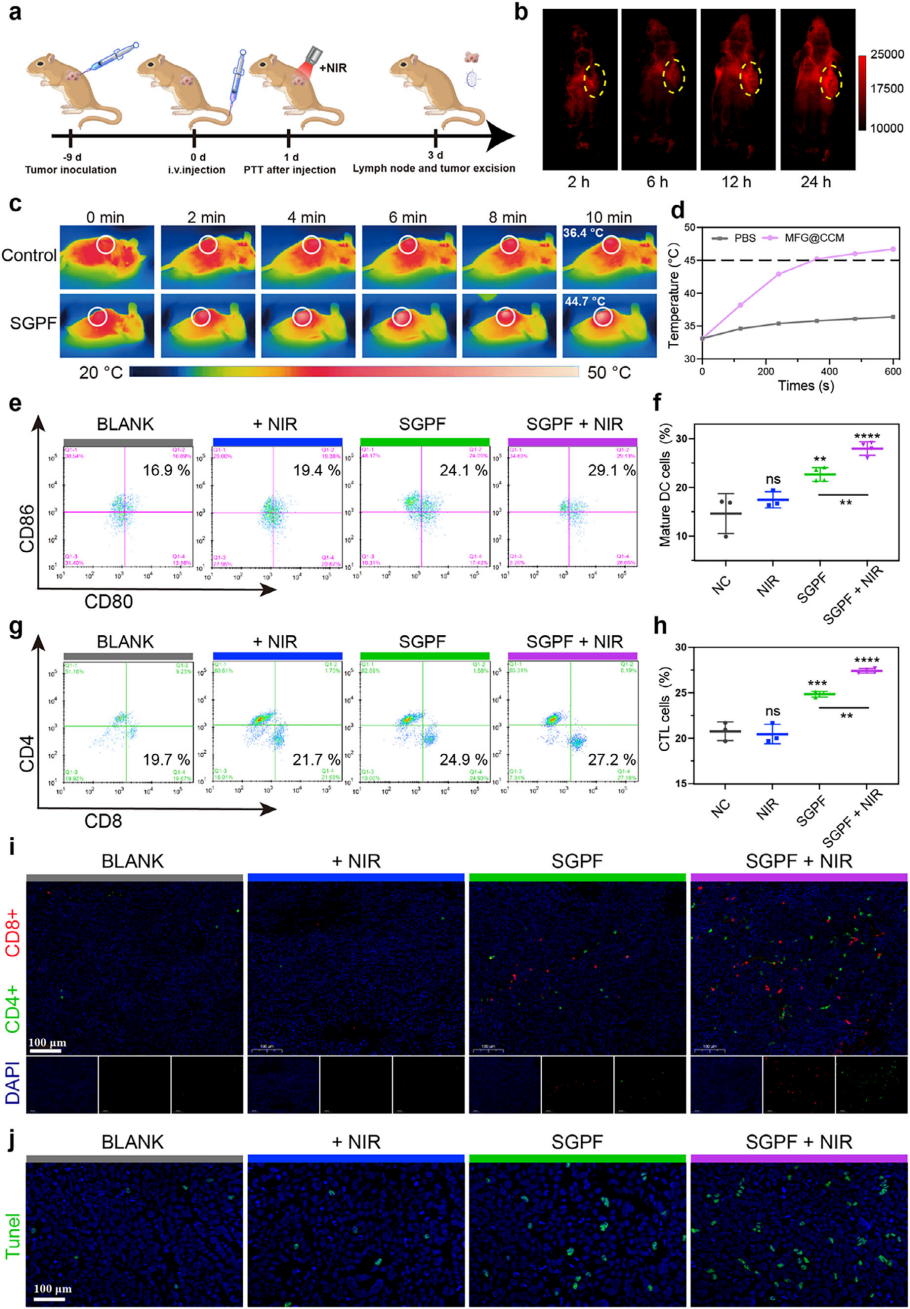
In vivo multimodal imaging and early‐stage immune activation of SGPF‐mediated phototherapy. (a) Experimental design timeline for in vivo studies. (b) Time‐dependent tumor accumulation of SGPF monitored by fluorescence imaging over 24 h post‐injection. (c) The representative thermal images of the mice with intravenous administration of PBS and SGPF under NIR irradiation for 10 min (1.5 W/cm^2^). (d) The corresponding temperature changes over time curve of tumor regions in (c). (e) Representative flow cytometry plots showing mature dendritic cells (CD86^+^/CD80^+^, gated on CD11c^+^ cells) in tumor‐draining lymph nodes 3 days post‐treatment with: PBS, NIR alone, SGPF alone, or SGPF + NIR (n = 3). (f) Quantification of mature DC frequency from (e) (n = 3). (g) Flow cytometry analysis of tumor‐infiltrating CD8^+^ and CD4^+^ T cells under the indicated treatments. (h) Quantitative assessment of T cells from (g) (n = 3). (i) Immunofluorescence imaging of tumor sections stained for CD8^+^ (red) and CD4^+^ (green) T cells. Nuclei counterstained with DAPI (blue). (j) TUNEL assay in tumor tissues following treatments. Data are mean ± SD. Scale bars: (i) 100 µM; (j) 100 µM. ns indicated no significance, ^*^ *p* < 0.05, ^**^ *p* < 0.01, ^***^ *p* < 0.001 versus control group.

The SGPF nanosystem demonstrated exceptional fluorescence imaging capabilities, photothermal/photodynamic performance, and immunostimulatory effects in material characterization and cellular assays. To evaluate its efficacy for intraoperative imaging and antitumor immunity in vivo, C3H mice bearing LM8 subcutaneous tumors were subjected to various treatments. Tumor tissues, draining lymph nodes (dLNs), and spleens were harvested 72 h post‐treatment for early immune activation assessment (Figure 9a). Following intravenous SGPF administration, NIR‐IIb imaging revealed progressive tumor accumulation of AIE molecules within 2 h, achieving optimal signal‐to‐noise ratios at 12–24 h (Figure 9b). Organ imaging demonstrated the preferential accumulation of the SGPF at the tumor site (Figure S17), thereby satisfying requirements for fluorescence‐guided surgical resection. Thermal imaging (Figure 9c, d) was applied to record the temperature rise curve of the OS tumor at irradiation of NIR, demonstrating the mild temperature PTT of SGPF (approximately 45°C). In this study, the 808 nm laser serves solely for preoperative imaging and tumor delineation, while the 1064 nm laser is specifically utilized for therapeutic intervention. Although mild PTT has been shown to enhance the “hot” state of the TME, some studies suggest that even a small increase in temperature can trigger the upregulation of HSP90 or PD‐L1 [69, 70]. This upregulation serves as a self‐protective mechanism, which ultimately leads to immunosuppression. Systemic immune activation was further corroborated by elevated serum concentrations of proinflammatory cytokines (IFN‐γ, IL‐6, TNF‐α) in the combined treatment group (Figure S18). Flow cytometry analysis of bone marrow‐derived dendritic cells (BMDCs) demonstrated significantly increased maturation in SGPF‐treated mice, evidenced by upregulated surface expression of CD11c^+^ CD80^+^ CD86^+^ markers relative to controls (Figure 9e, f). This effect was potentiated by NIR co‐treatment, attributable to enhanced tumoricidal activity. The spleen, serving as the primary immunological hub, facilitated MHC‐I/II‐dependent activation of tumor‐specific CD8^+^/CD4^+^ T cells, triggering clonal expansion within 0–72 h [71]. SGPF+NIR treatment markedly increased proliferating CD8^+^ and CD4^+^ T cells in the spleen (Figure 9g, h). This expansion is mechanistically significant: CD8^+^ T cells acquire augmented tumoricidal capacity, while CD4^+^ Th1 cells secrete IFN‐γ/IL‐2 to sustain antigen‐presenting cell (APC) activation and counteract TME immunosuppression.

Multiplex immunofluorescence of tumor sections confirmed substantial infiltration of cytotoxic T lymphocytes (CTLs) and T helper cells in the SGPF+NIR cohort (Figure 9i), indicative of a transition from an “immune‐cold” to “immune‐hot” TME. TUNEL^+^ staining—a biomarker of ICD—was significantly elevated in this group (Figure 9j). This positivity correlated with DAMP release (e.g., calreticulin exposure and HMGB1 translocation), which drives dendritic cell maturation, antigen cross‐presentation, and CD8^+^ T cell priming. Subsequent immunofluorescence validated pronounced CRT externalization in SGPF+NIR‐treated tumors (Figure S19).

Concurrently, suppressed tumor proliferation was evidenced by reduced Ki‐67 expression and aberrant histopathology in treatment groups (Figures S20 and S21). Histopathological and serological analyses performed 3 days post‐treatment revealed no evidence of acute toxicity. Hematoxylin and eosin (H&E)‐stained sections of major organs (heart, liver, spleen, lungs, kidneys) showed no structural abnormalities (Figure S22), while complete blood counts and biochemical profiles indicated the absence of hemolysis, hepatotoxicity, or nephrotoxicity (Figure S23). The favorable biosafety profile of SGPF is attributable to its fully organic composition, which precludes toxic metal ion accumulation and enables metabolic clearance, highlighting its translational potential for clinical oncology.

### 3.8 Suppression of Distant Metastases and Alleviation of Immunosuppressive Tumor Microenvironment In Vivo

**FIGURE 10:**
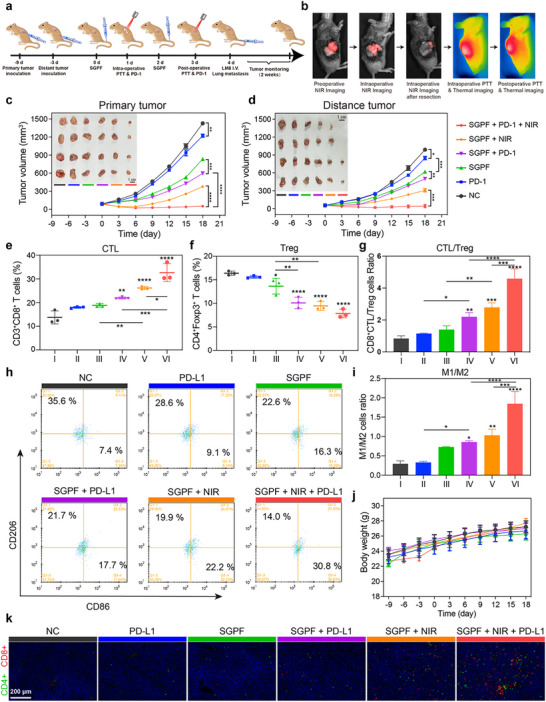
SGPF‐mediated surgical resection combined with intraoperative and postoperative phototherapy sensitize OS to ICB immunotherapy by remodeling the immune microenvironment against primary/distant tumors and metastasis. (a) Experimental design: Surgical resection of primary OS with intraoperative/postoperative SGPF‐mediated phototherapy combined with anti‐anti‐PD‐1 antibody immunotherapy to control primary and distant tumors, and prevent lung metastasis. (b) In vivo fluorescence and thermal imaging in a subcutaneous osteosarcoma mouse model. (c, d) Growth kinetics of (c) primary and (d) distant tumors across treatment groups (n = 4). (e, f) Flow cytometry analysis of immunosuppressive regulatory T cells (Treg) (CD4^+^ FoxP3^+^ CD25^+^) and Cytotoxic T lymphocyte (CTL) (CD3^+^CD8^+^) cells in distant tumors (n = 3). (g) Quantitative CTL/Treg ratios from (e, f). Higher ratios indicate enhanced anti‐tumor immunity (n = 3). (h, i) Macrophage polarization analysis: (h) Representative flow plots of M1 (CD80^+^) and M2 (CD206^+^) phenotypes; (i) M1/M2 ratio quantification (n = 3). (j) Body weight changes in C3H mice during different treatment (n = 4). (k) Immunofluorescence of CD8^+^ (red) and CD4^+^ (green) T cells in distant tumors. Nuclei: DAPI (blue). Data are mean ± SD. Scale bars: (c, d) 1 cm; (k) 200 µM; ns indicated no significance, ^*^ *p* < 0.05, ^**^ *p* < 0.01, ^***^ *p* < 0.001 versus control group.

Patients with advanced OS—particularly those with axial non‐extremity tumors refractory to first‐ and second‐line chemo/radiotherapy—exhibit significantly poorer prognoses than extremity sarcoma counterparts [72]. Clinical management remains challenging due to anatomical constraints: extensive pelvic tumors invading the aorta or spinal lesions compromising cord integrity preclude radical resection, as such procedures risk fatal hemorrhage or paralysis. While palliative debulking alleviates symptoms, residual disease inevitably progresses, underscoring an urgent need for integrated strategies combining intraoperative imaging‐guided resection with sustained perioperative therapy to mitigate recurrence and metastasis. To model this clinical scenario, C3H mice were implanted with LM8 cells subcutaneously in the right flank (‐9 days) to establish primary tumors and in the left flank (‐3 days) to simulate metastatic deposits (Figure 10a). As shown in Figure 10b, 24 h after initial intravenous SGPF administration, partial tumor resection was performed under fluorescence guidance, leaving residual masses of approximately 50 mg. In the combination group, intraoperative NIR irradiation was applied prior to wound closure, followed by repeat SGPF injection and NIR exposure on days 2 and 3. anti‐PD‐1 antibody blockade cohorts received anti‐PD‐1 antibody intraperitoneally on days 1 and 3. To assess metastatic suppression, LM8 cells were intravenously injected on day 4 to seed pulmonary colonies, with endpoint analyses conducted on day 18.

Notably, anti‐PD‐1 antibody monotherapy demonstrated limited efficacy against both primary and simulated metastatic lesions (Figure 10c, d). Conversely, PD‐L1 blockade substantially potentiated the antitumor effects of SGPF alone and SGPF‐phototherapy combinations. Immediate volume reduction was observed in phototherapy groups post‐irradiation, confirming SGPF‐mediated primary tumor suppression. The SGPF+NIR cohort exhibited robust primary tumor growth inhibition and abrogated development of simulated metastases, indicating systemic immune activation. Immunoprofiling of metastatic lesions revealed significant microenvironmental remodeling: CTLs and T helper cell infiltration were markedly increased while Treg populations decreased, yielding an elevated CTL/Treg ratio (Figure 10e–g; Figures S24 and S25). This shift is clinically consequential—heightened CTL/Treg ratios reverse effector T cell suppression by diminishing Treg‐mediated immunosuppression (e.g., IL‐10/TGF‐β reduction), enhance tumor‐specific cytotoxicity, and improve T cell infiltration depth. As evidenced clinically, pre‐treatment CTL/Treg elevation correlates with higher anti‐PD‐1 antibody response rates and more progression‐free survival gains [73, 74]. Macrophage polarization toward an immunostimulatory M1 phenotype was concurrently observed in SGPF+NIR‐treated metastases (Figure 10h, i; Figure S26), signifying transition from immunosuppressive to immune‐permissive microenvironments. No significant body weight variations were detected across groups (Figure 10j), corroborating the biocompatibility of combined therapy. Multiplex immunofluorescence in Figure 10k confirmed intensified CTL/Th infiltration in primary tumors of the SGPF+NIR cohort, establishing an immune‐“hot” milieu. anti‐PD‐1 antibody co‐administration further amplified T cell trafficking, explaining the near‐complete suppression of metastatic outgrowth. While this study demonstrates that SGPF‐mediated phototherapy and TME remodeling successfully activate a potent anti‐tumor immune response, resulting in significant inhibition of established primary and metastatic lesions, it primarily addresses the therapeutic goal of treating existing disease. The important question of whether this treatment can elicit a long‐term immunological memory, capable of preventing the outgrowth of newly introduced tumor cells (as evaluated in a tumor rechallenge model), remains open. Such an investigation, which aligns more closely with the paradigm of prophylactic cancer vaccines, represents a distinct and valuable direction for future research [67]. Exploring the vaccine‐like potential of our platform, possibly by combining it with strategies to amplify antigen persistence and memory T‐cell differentiation, will be a critical next step in evaluating its potential for achieving durable, systemic anti‐tumor immunity and preventing recurrence.

### 3.9 SGPF‐based Immunotherapy Inhibits Lung Metastasis of Osteosarcoma

Pulmonary metastasis represents a critical determinant of clinical outcomes in OS progression, serving as the most frequent hematogenous dissemination site. Its presence precipitously reduces 5‐year survival rates from 60–70% in non‐metastatic patients to <20%, establishing metastatic lung disease as the primary cause of OS‐related mortality [75]. Immunotherapeutic strategies demonstrate potential for metastasis prevention through tumor microenvironment remodeling [76]. Dual anti‐PD‐1 antibody/CTLA‐4 blockade achieves long‐term disease‐free survival in murine models, significantly outperforming monotherapies. Photothermal immunocomplexes concurrently suppress primary tumor growth and distant metastasis by activating systemic antitumor immunity. Neoantigen‐targeted approaches inhibit metastatic potential, offering promising clinical translation avenues. Collectively, pulmonary metastasis constitutes the principal therapeutic bottleneck in OS, while combinatorial immunotherapies hold promise for altering its inherently metastatic natural history.

**FIGURE 11:**
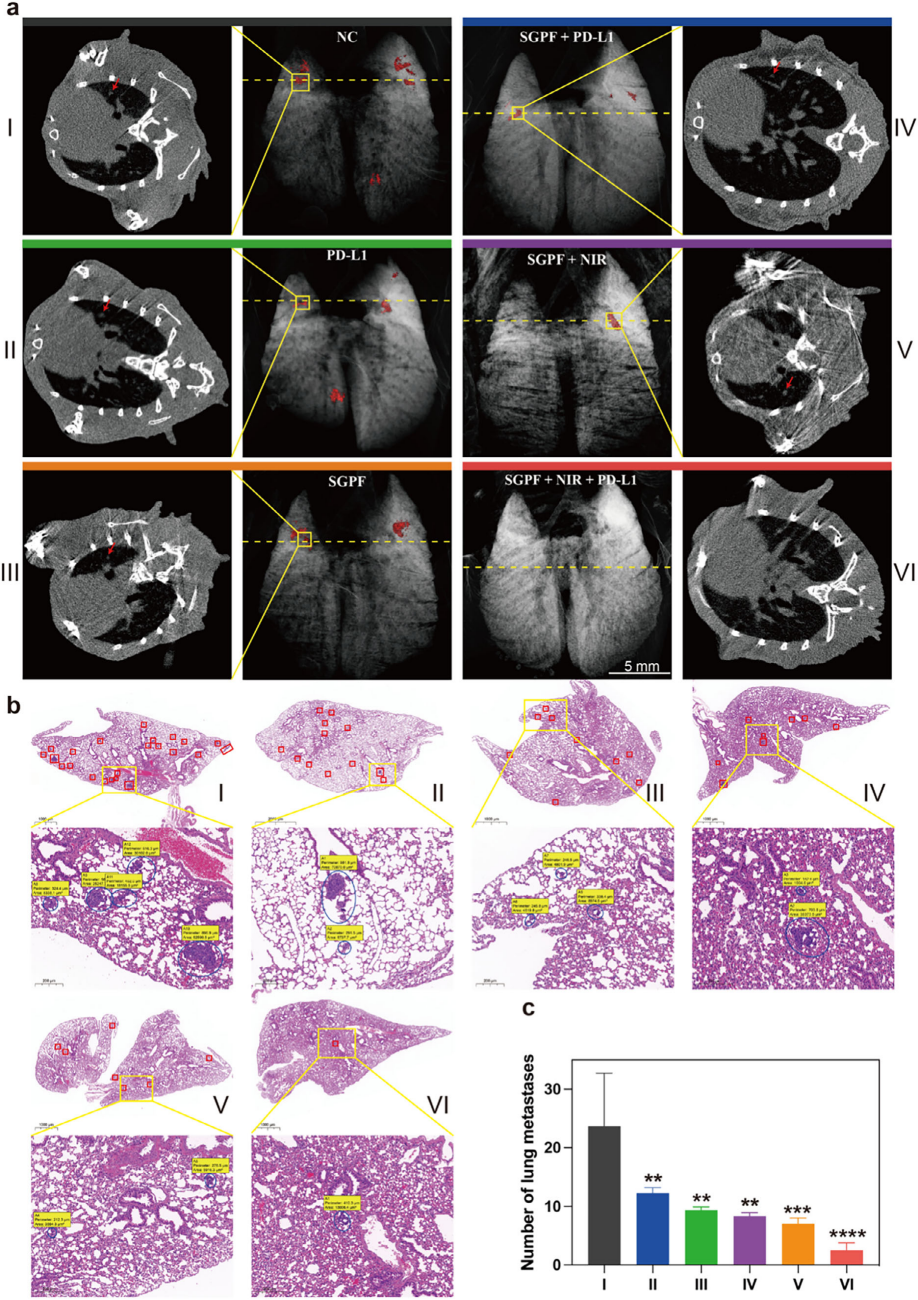
Inhibitory effect of sGPF‐mediated immunosensitizing therapy on lung metastasis in osteosarcoma. (a) Micro‐CT imaging of pulmonary metastases 14 days post‐intravenous LM8 injection in treatment groups from Figure 9a. Arrows indicate lesions. (b) Representative H&E‐stained lung sections showing micrometastases at study endpoint. (c) Quantification of metastatic foci per lung section (n = 3). Scale bars: (a) 5 mm; (b) 1000 µM (overview) and 200 µM (magnification). Data are mean ± SD. ns indicated no significance, ^**^ *p* < 0.01, ^***^ *p* < 0.001, ^***^ *p* < 0.0001 versus control group.

In our experimental metastasis model, LM8 cells were intravenously injected to establish pulmonary colonies. In‐vivo CT imaging prior to endpoint analysis revealed multiple macrometastases (1–2 mm diameter) in control and monotherapy groups, whereas combination treatments markedly reduced detectable lesions (Figure 11a). Notably, no CT‐discernible nodules were observed in the SGPF+phototherapy+anti‐PD‐1 antibody cohort. Given CT's resolution limitations for micrometastases, lung sections underwent histopathological evaluation. Micrometastatic foci (50–200 µM) were quantified per section, confirming significantly higher burdens in controls (>2 fold increase) versus all treatment arms (Figure 11b, c). The SGPF+phototherapy+anti‐PD‐1 antibody group exhibited near‐complete suppression, with minimal micrometastasis counts (as low as 1 focus per section), validating systemic immune activation as a viable strategy for preventing OS pulmonary dissemination.

Beyond demonstrating the individual functions of its components, a critical analysis reveals that the SGPF nanoplatform achieves a therapeutic outcome that transcends mere functional complementarity, approaching a state of profound synergistic amplification. This synergy is orchestrated by the ALP‐responsive design, which ensures spatiotemporal coordination of the therapeutic actions. The enzymatic trigger does not merely release payloads; it initiates an interdependent cascade: the concurrent release and activation of the AIEgen for enhanced PTT/PDT, the vaporization of PFH to alleviate hypoxia, and the liberation of Ganetespib to suppress glycolysis. These processes are interwoven into positive feedback loops. For instance, the phototherapy‐induced immunogenic pyroptosis releases tumor antigens, while Ganetespib‐mediated downregulation of HK2/PKM2 dismantles lactate‐driven immunosuppression. This dual action does not occur in parallel but synergistically converts an immunologically “cold” tumor microenvironment into a “hot” one, dramatically enhancing T‐cell infiltration and activation. Furthermore, the components reciprocally overcome each other's limitations—the generation of hypoxia‐insensitive Type‐I ROS maintains efficacy under initial low oxygen tension, which is subsequently potentiated by PFH‐derived oxygen release. Therefore, the imaging, ablation, hypoxia relief, and metabolic reprogramming capabilities are mechanistically interlocked. This integrated cascade creates a systemic anti‐tumor immune response with efficacy greater than the sum of its parts, enabling effective control of both primary and metastatic lesions in advanced osteosarcoma.

## 4 Conclusion

This study establishes an ALP‐responsive theranostic nanoplatform (SGPF) that addresses the critical challenge of unresectable advanced osteosarcoma through integrated imaging‐guided resection and multimodal therapy, where molecular engineering for constructing selenium‐incorporated AIEgen (STEA) with narrowed HOMO‐LUMO gaps and enhanced intramolecular torsion enables deep tissue NIR‐IIb emission for precision surgery while simultaneously generating hypoxia‐insensitive type‐I ROS and hyperthermia under 1064 nm irradiation. Concurrently, the platform orchestrates spatiotemporal therapeutic synergy wherein ALP‐triggered PFH vaporization reverses hypoxia to potentiate caspase‐3/GSDME‐mediated pyroptosis and immunogenic cell death, while co‐delivered Ganetespib suppresses glycolytic flux via HK2/PKM2 downregulation to ablate lactate‐driven acidosis and dismantle immunosuppressive barriers, collectively converting immunologically “cold” tumors into active niches. Ultimately, this paradigm unifies palliative resection accuracy, in situ residual eradication, and systemic immunity activation to achieve unprecedented control of primary/metastatic lesions, thereby redefining care standards for neurovascular‐invaded osteosarcoma and offering a blueprint for matrix‐compacted, immunotherapy‐resistant solid tumors.

## Conflicts of Interest

The authors declare no conflict of interest.

## Supporting information




**Supporting File**: advs73735‐sup‐0001‐SuppMat.docx.

## Data Availability

The data that support the findings of this study are available from the corresponding author upon reasonable request.
