# Supplementary material for: Alkaline Phosphatase‐Activated NIR‐II AIEgens Nanosystem for Surgical and Postoperative Closed‐Loop Therapy of Advanced Osteosarcoma
Source: Adv Sci (Weinh). 2026 Jan 20;13(16):e16035. doi: 10.1002/advs.202516035 (PMC13042677; doi:10.1002/advs.202516035)
Supplement: Supplementary file 1 — Supporting File: advs73735‐sup‐0001‐SuppMat.docx. [file ADVS-13-e16035-s001.docx]

Supporting Information

Alkaline Phosphatase-Activated NIR-II AIEgens Nanosystem for Surgical and Postoperative Closed-Loop Therapy of Advanced Osteosarcoma

Kaiyuan Liu^1,2, †^, Ruotong Li^1, †^, Li Zhang^3, †^, Hengli Lu^2^, Binhui Yang^2^, Qian Hu^1^, Yining Tao^2^, Haoran Mu^2^, Jing Han^2^, Pengfei Zan^2^, Jiakang Shen^2^, Dongqing Zuo^2^, Hongsheng Wang^2, *^, Wei Sun^2, *^, Xingjun Zhu^1, *^

R. Li, Q. Hu, X. Zhu

School of Physical Science and Technology & State Key Laboratory of Advanced Medical Materials and Devices

ShanghaiTech University, 393 Middle Huaxia Road

Shanghai, 201210, P.R. China

E-mail: zhuxj1@shanghaitech.edu.cn

1. Liu, H. Lu, B. Yang, Y. Tao, H. Mu, J. Han, P. Zan, J. Shen, D. Zuo, H. Wang, W. Sun

Department of Bone Tumor Surgery, Shanghai General Hospital

School of Medicine, Shanghai Jiao Tong University,

Shanghai 200080, P. R. China.

Email: wanghongsheng@sjtu.edu.cn

E-mail: viv-sun@sjtu.edu.cn

L. Zhang

Department of Dermatology, Shanghai Key Laboratory of Molecular Medical Mycology, Shanghai Changzheng Hospital, Naval Medical University,

Shanghai 200003, P.R. China

† These authors contributed equally to this work.

**Synthesis of STEA**


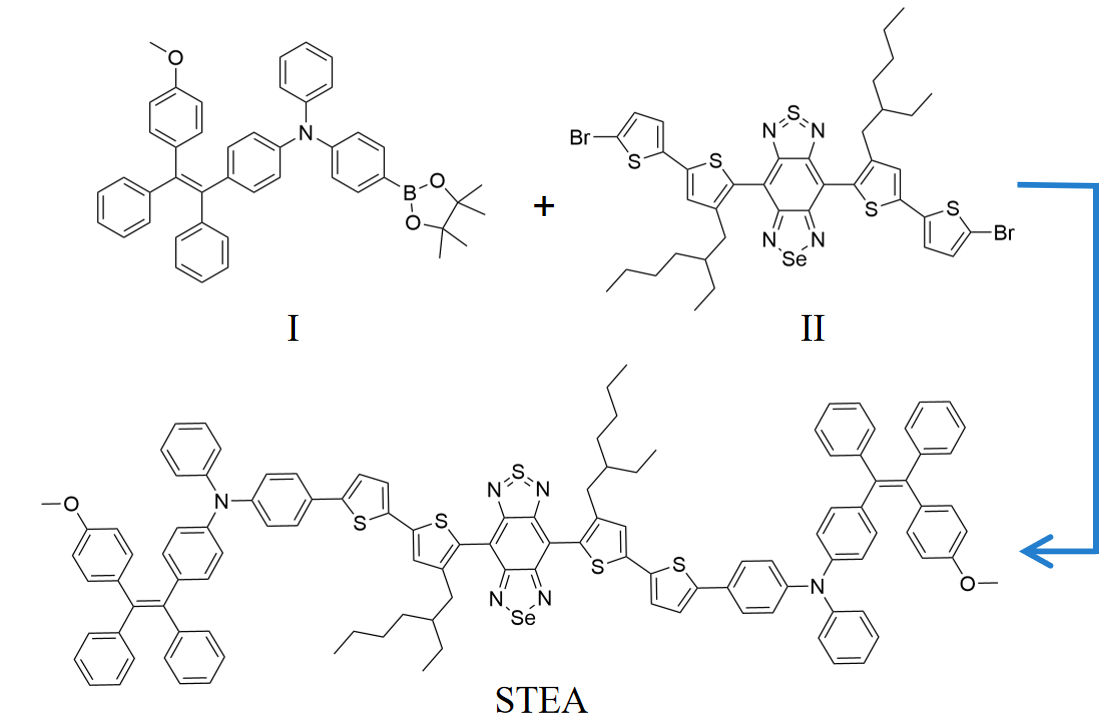


**Scheme S1.** Synthetic routes to STEA.

Compounds I and II were provided by the AIE Institute, South China University of Technology. 2.2 equivalents of compound I, 1 equivalent of compound II, 0.015 equivalents of Pd[P(t-Bu)₂(4-NMe₂-Ph)]Cl₂ (Pd132), and 0.3 equivalents of potassium carbonate were dissolved in 2 mL of toluene and 0.5 mL of methanol. The reaction mixture was stirred at 90 °C under nitrogen protection for 1.0 hour. After the reaction, the mixture was diluted with 40 mL of dichloromethane (DCM) and passed through a thin silica gel layer. The silica was rinsed with DCM, and the combined filtrate was concentrated. The crude product was purified by column chromatography using DCM/petroleum ether (PE) in a gradient from 1:2 to 1:1 as the eluent, yielding a dark brown solid. The same chromatography procedure was repeated twice to obtain 0.49 g of dark brown powder with approximately 90% purity by TLC and a yield of 75.7%. Further washing with n-hexane under sonication afforded 0.22 g of brown powder with over 95% TLC purity and a yield of 33.8%.

^1^H NMR (500 MHz, Methylene Chloride-d_2_) δ 7.40 – 7.18 (m, 7H), 7.18 – 6.86 (m, 43H), 6.79 (s, 5H), 6.67 (d, J = 8.3 Hz, 5H), 3.73 (s, 6H), 1.84 (s, 2H), 1.51 – 1.26 (m, 20H), 0.95 (t, J = 7.3 Hz, 6H), 0.89 (t, J = 7.1 Hz, 6H).

**Figure S1.** ^1^H NMR spectrum of STEA.

**
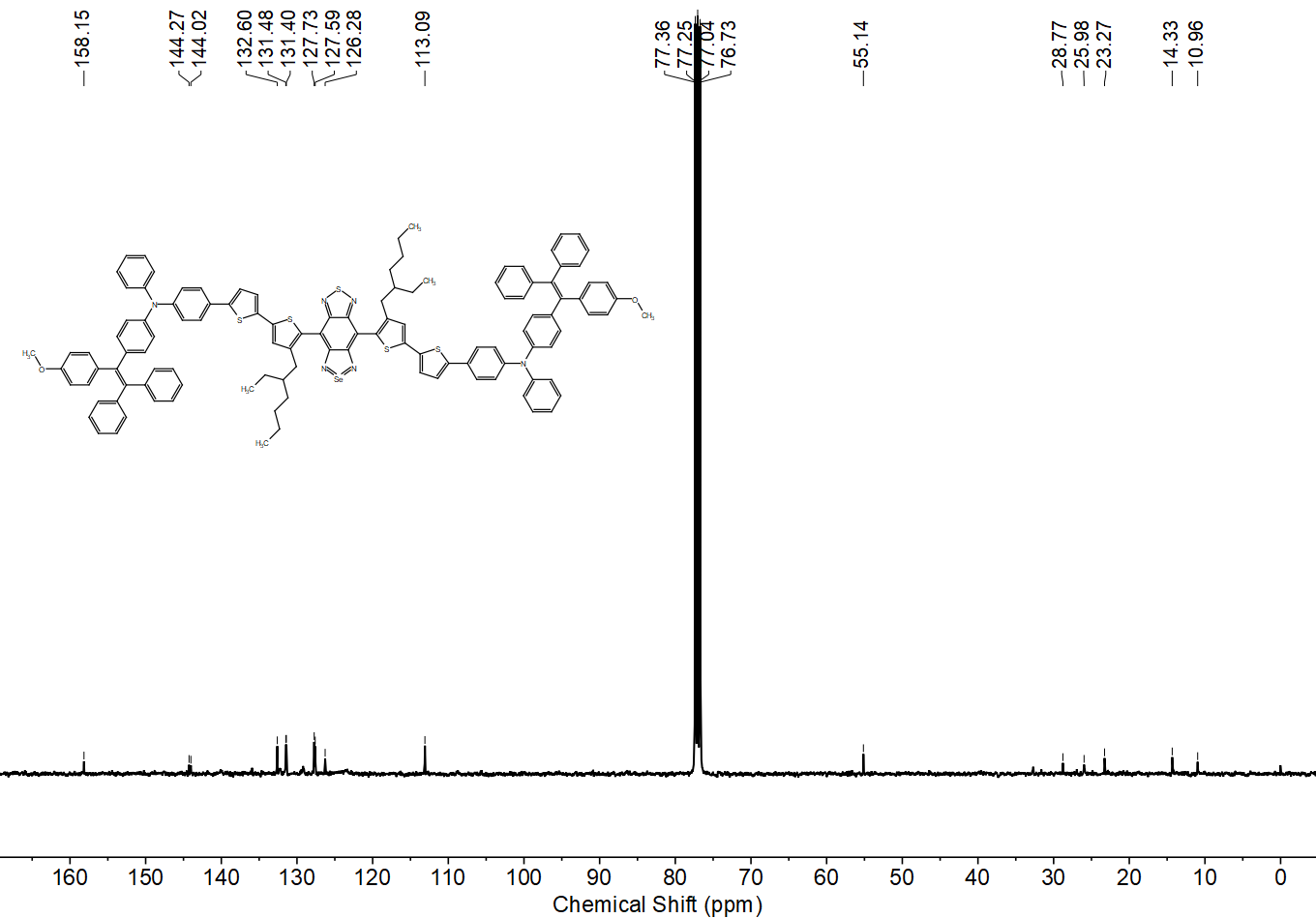
**

**Figure S2.** ^1^C NMR spectrum of STEA.


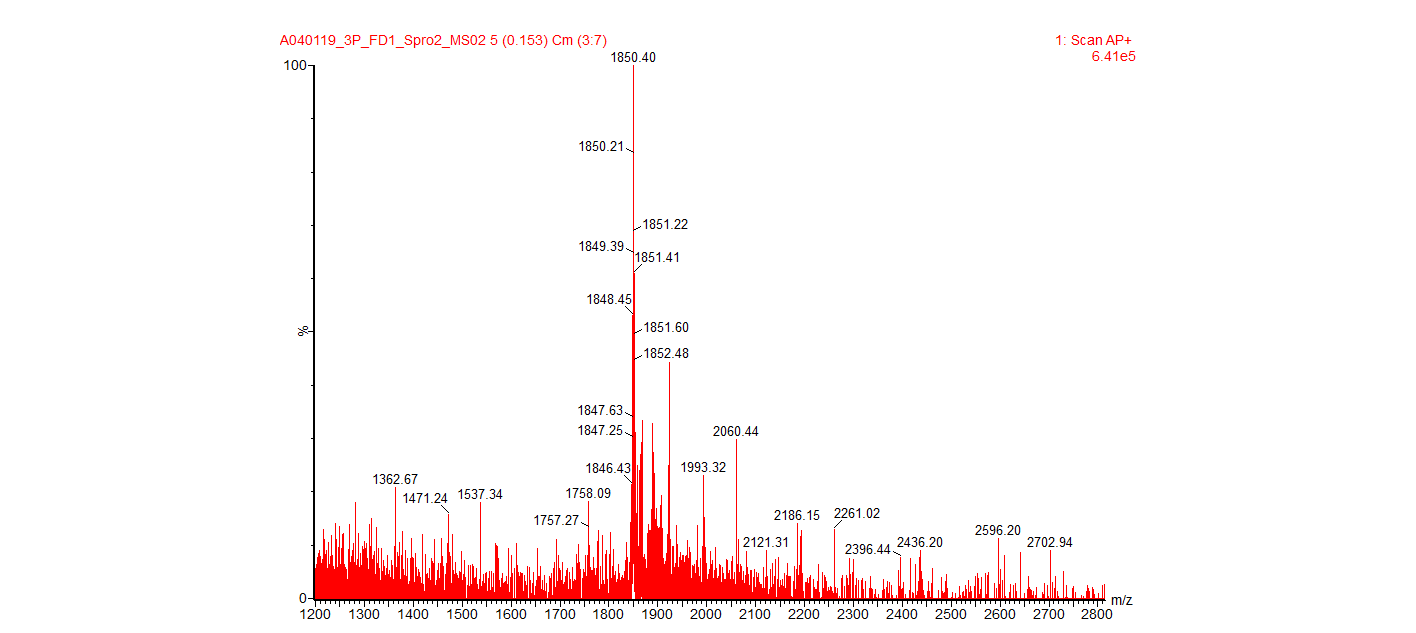


**Figure S3.** Mass spectrum of STEA.


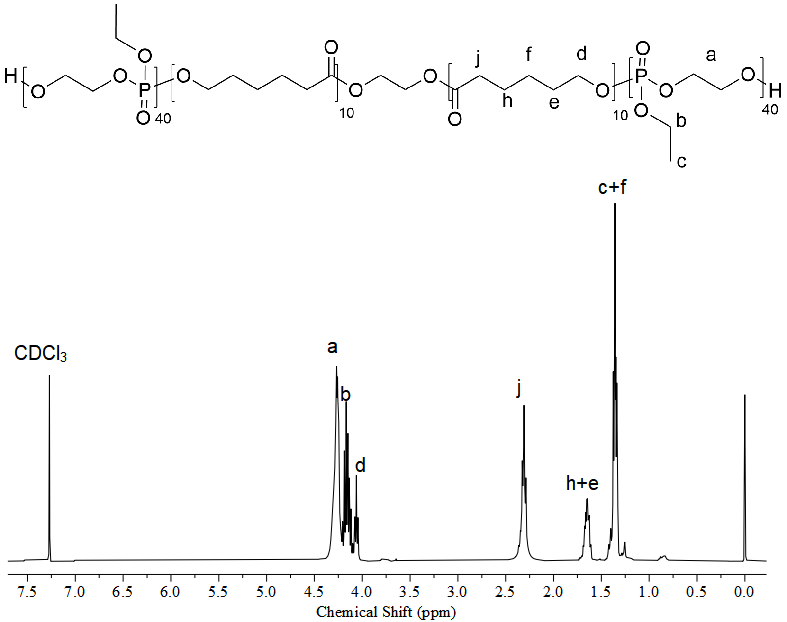


**Figure S4.** ^1^H NMR spectrum of PEEP.


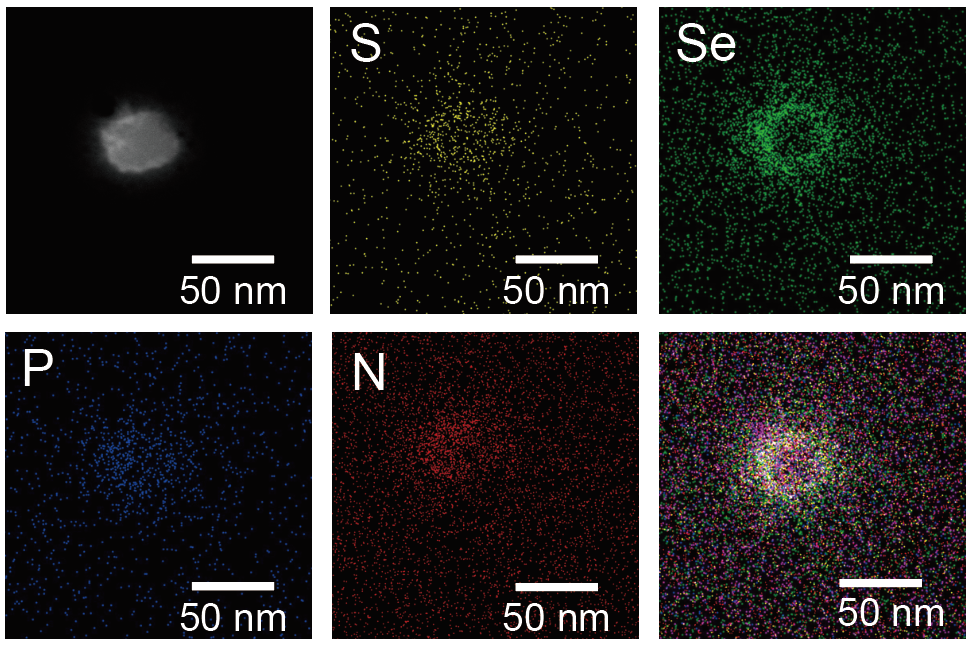


**Figure S5**. Energy dispersive X-ray spectroscopy (EDS) elemental mapping of SGPF micelle showing the distribution of S, Se, P and N elements.


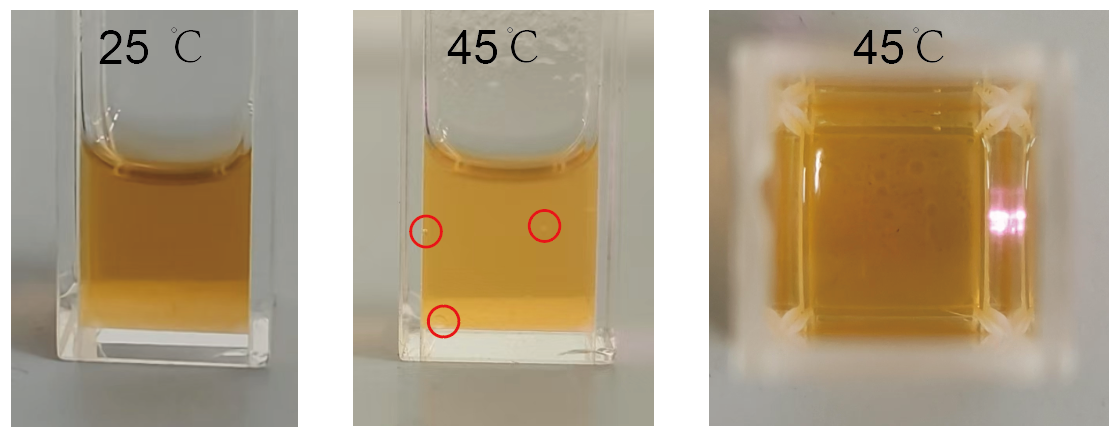


**Figure S6**.PFH undergoes vaporization during heating, releasing microbubbles.


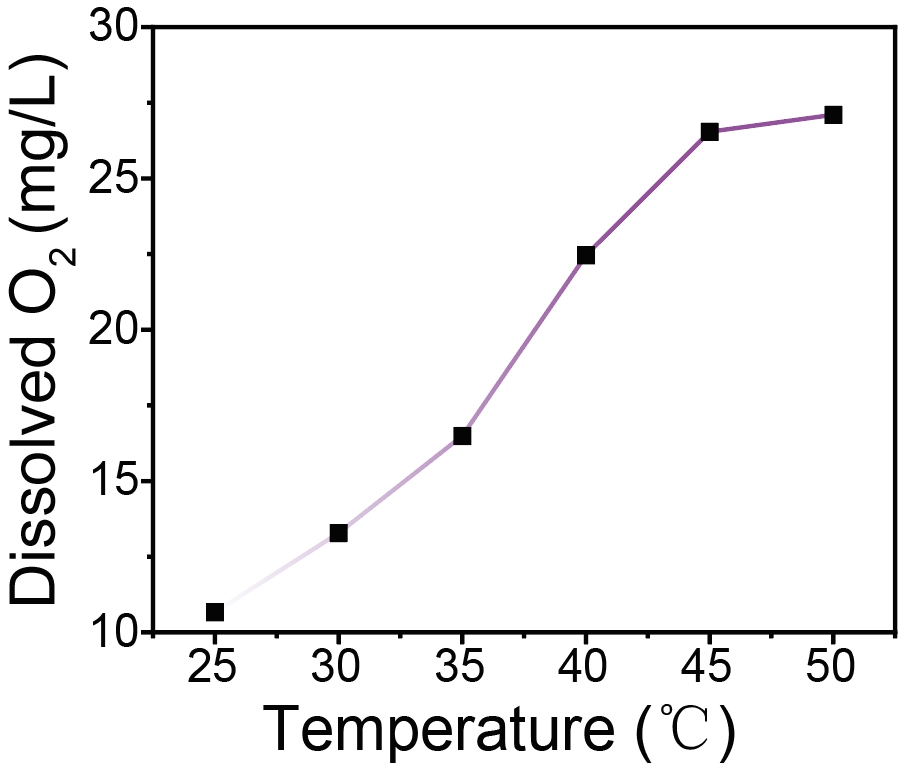


**Figure S7** The variation in oxygen content after heating.


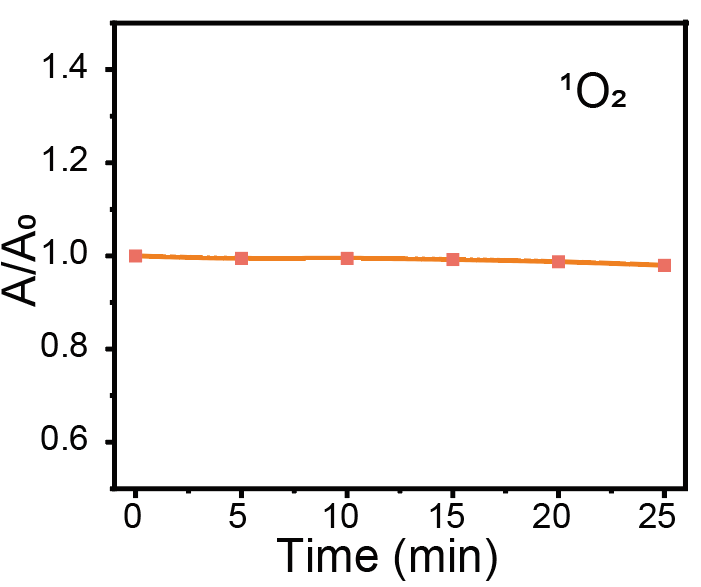


**Figure S8** Time-dependent absorption changes of ABDA indicating ^1^O₂ generation.


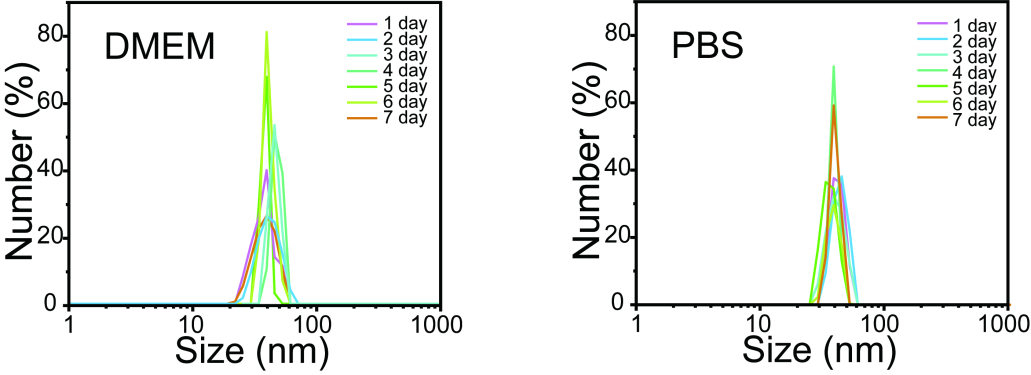


**Figure S9** The micellar size of SGPF dispersed in PBS and DMEM culture media over a period of 7 days.


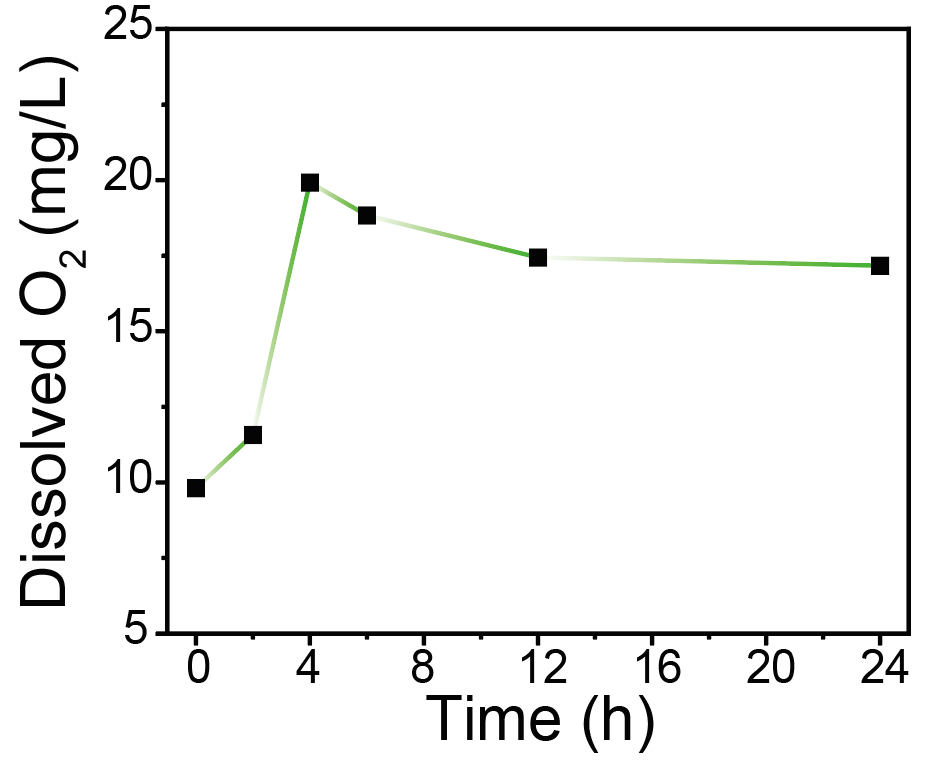


**Figure S10** The change in oxygen content after ALP-mediated decomposition of SGPF.


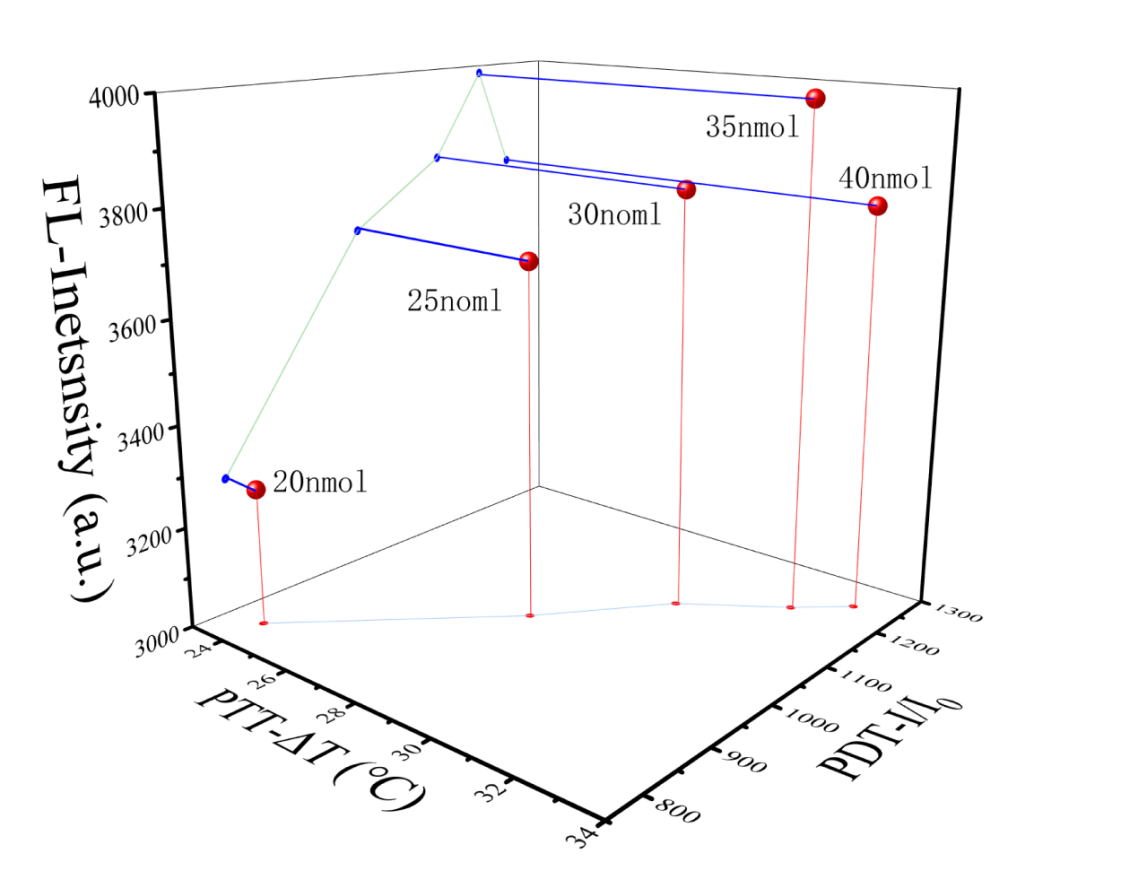


**Figure S11**. Statistical analysis of the photothermal therapy (PTT) and photodynamic therapy (PDT) effects, and fluorescence intensity of SGPF with different loading amounts of STEA under otherwise identical conditions, with all other variables controlled. (PTT and PDT effects, and fluorescence intensity of different SGPF were evaluated at the same STEA concentration of 15 μM. The PTT effect is presented as the temperature difference between the final and initial values, while the PDT effect reflects the ability of different groups to generate superoxide anions.)


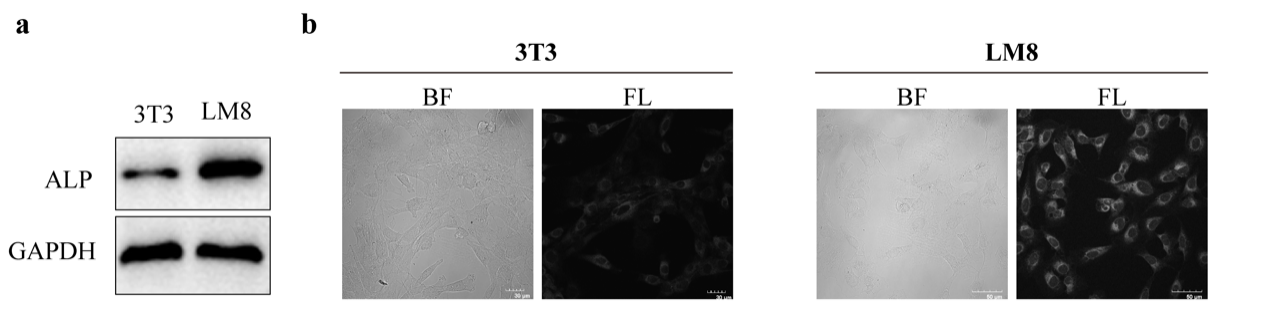


**Figure S12.** Cellular-level validation of the ALP-responsive selectivity of the SGPF nanoplatform. (a) Western blot analysis demonstrating differential expression of ALP in osteosarcoma LM8 cells compared to 3T3 cells. GAPDH serves as the loading control. (b) NIR fluorescence microscopy images of LM8 and 3T3 cells after 2-hours co-incubation with SGPF. The significantly brighter intracellular AIE fluorescence in LM8 cells (ALP-high) versus 3T3 cells (ALP-low) indicates selective, ALP-triggered release and cellular uptake of the AIEgen component.


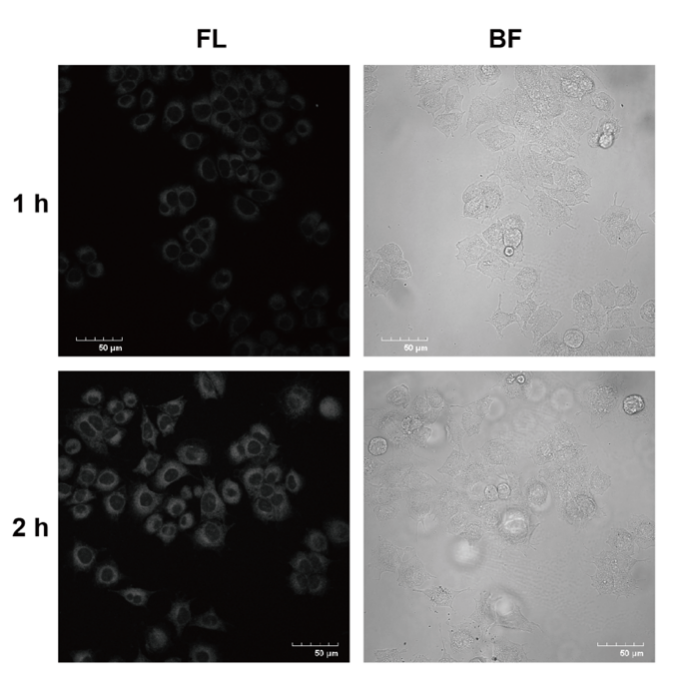


**Figure S13.** Near-infrared confocal images of AIE-labeled LM8 murine osteosarcoma cells following 1 or 2 hours incubation with SGPF nanoparticles.


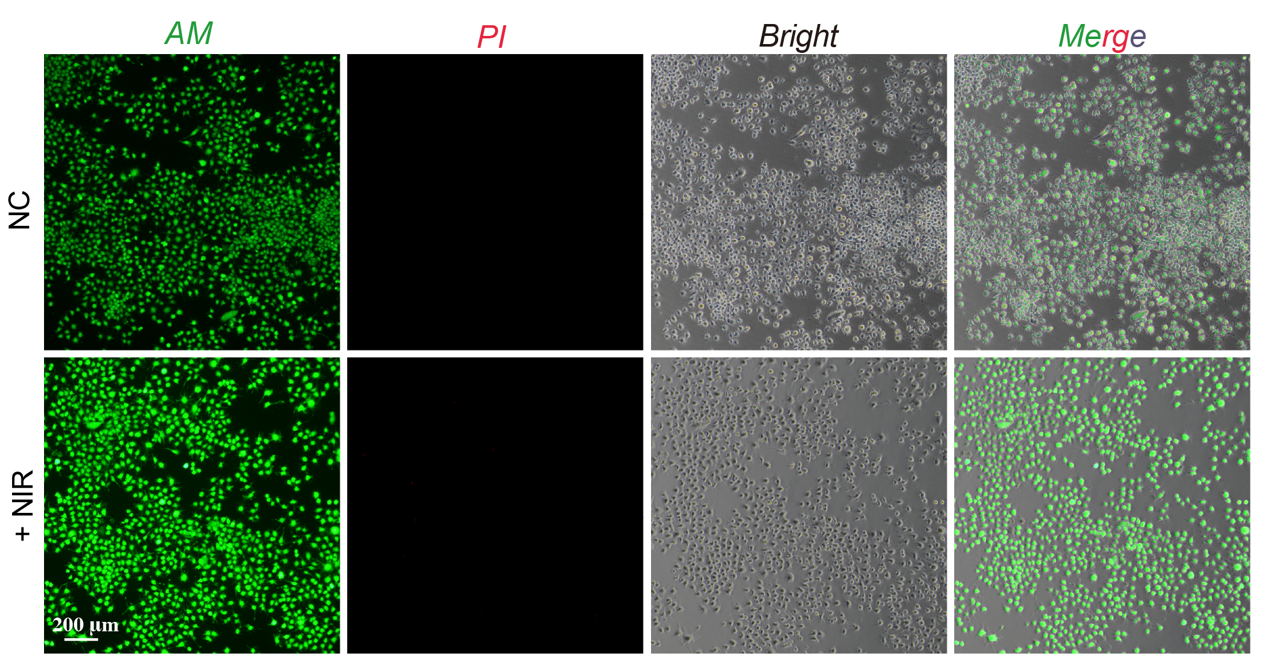


**Figure S14.** Viability of LM8 cells assessed by calcein-AM/PI live-dead staining with or without NIR irradiation (1064 nm, 1.5 W/cm², 10 min). Scale bars: 200 μm.


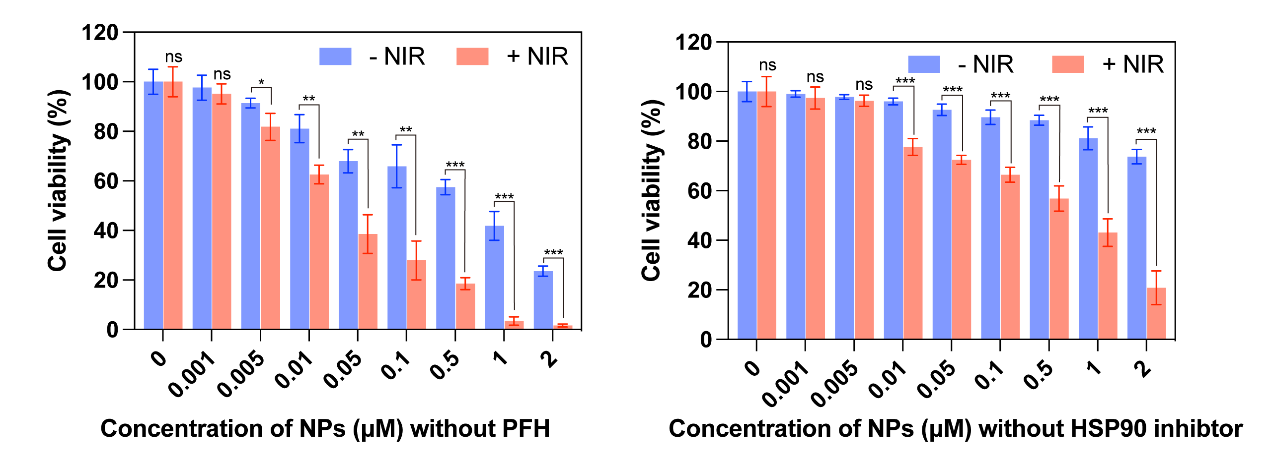


**Figure S15.** Cytotoxicity assessments of the SGPF lacking either PFH or Ganetespib, both with and without NIR irradiation (1064 nm, 1.5 W/cm^2^, 10 min).


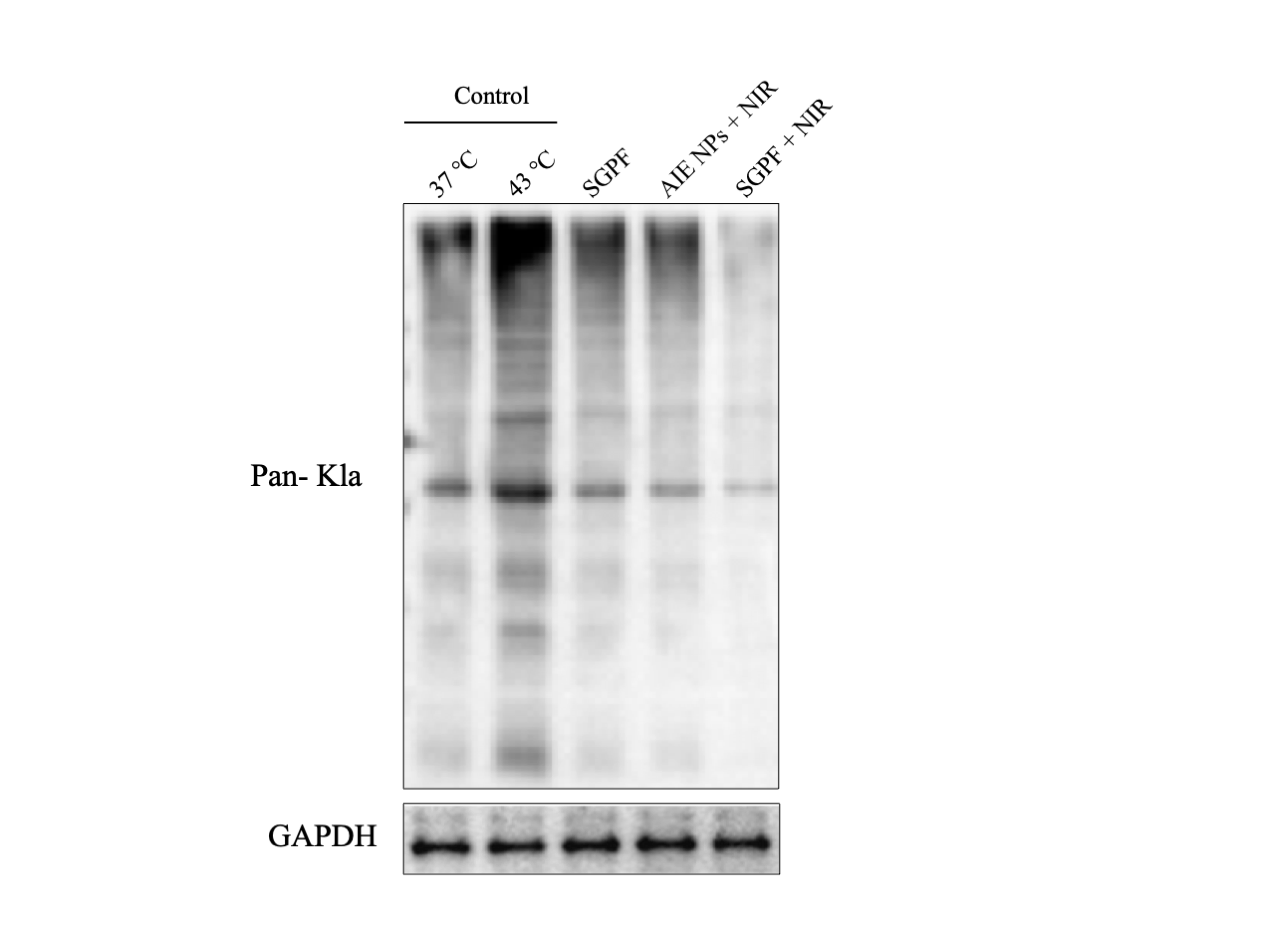


**Figure S16.** Western blot analysis of pan-lysine lactylation in LM8 cells maintained at 37°C or 43°C, and treated at 37°C with SGPF, AIE NPs plus NIR, or SGPF plus NIR.


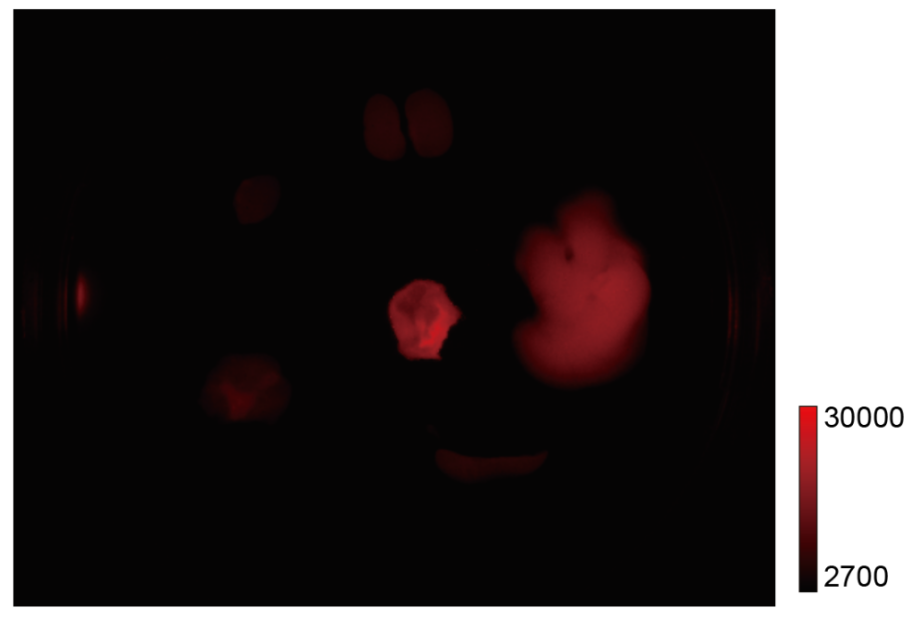


**Figure S17.** The *in-vitro* organ imaging of SGPF 36 h after its injection into the mouse.


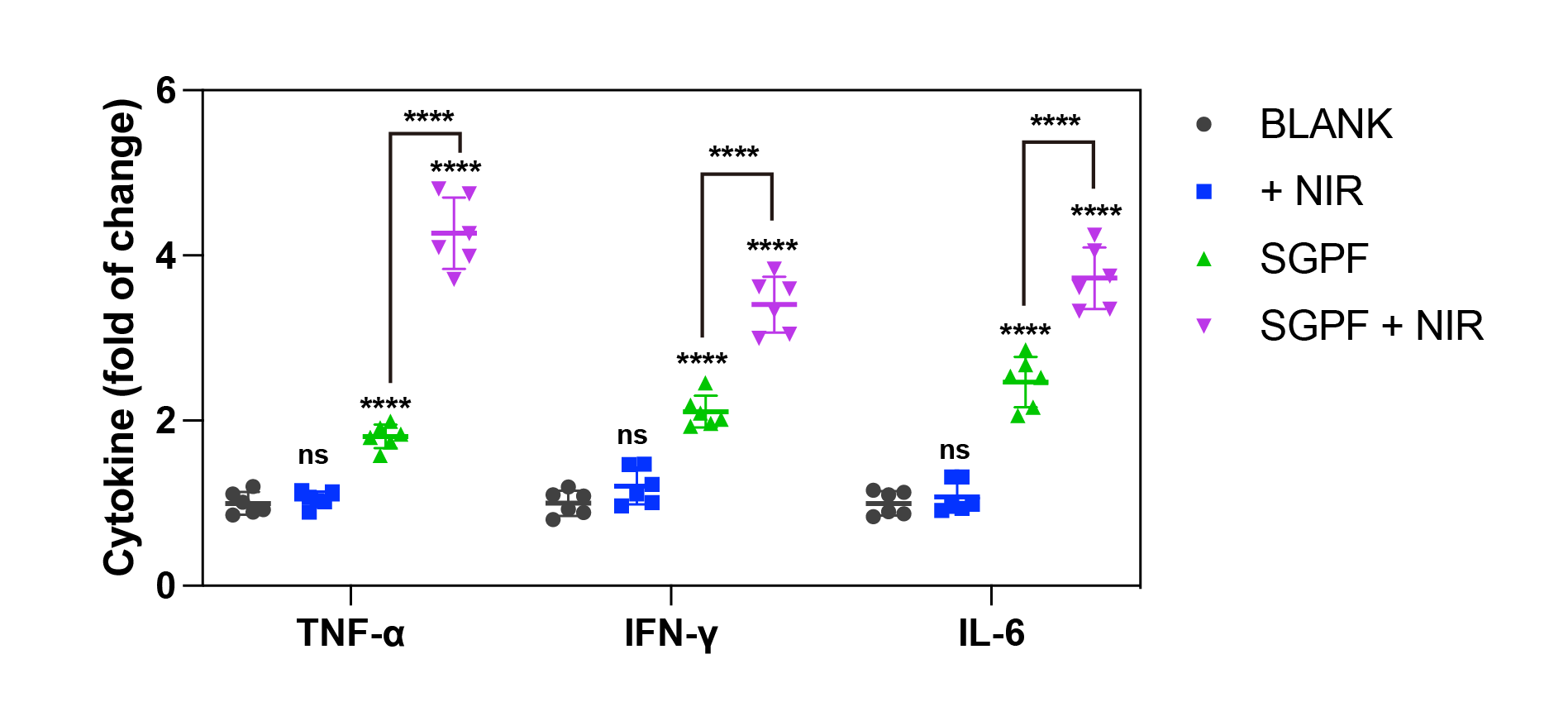


**Figure S18.** Serum concentrations of IFN-γ, TNF-α, and IL-6 cytokines 72 h post-treatment with PBS, NIR alone, SGPF alone, or SGPF plus NIR.


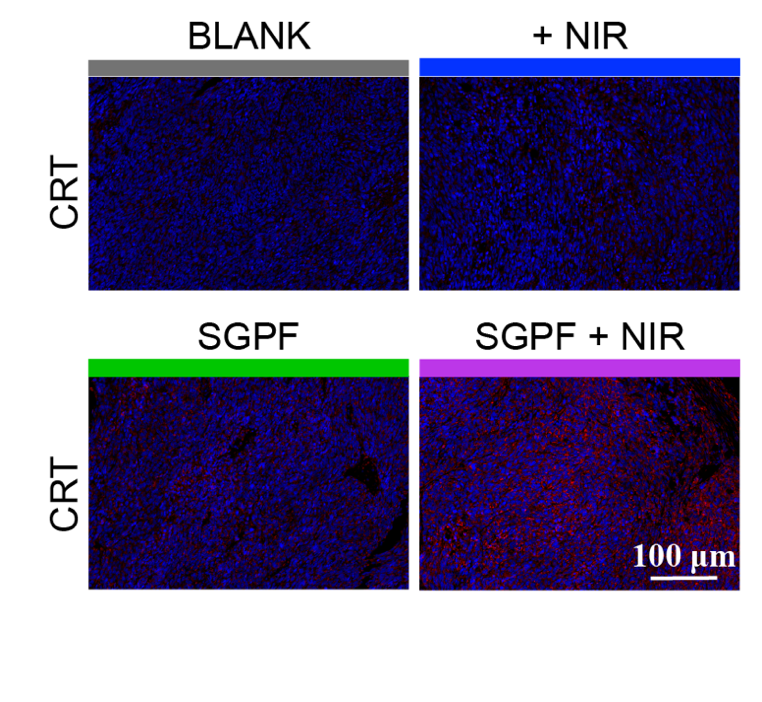


**Figure S19.** Immunofluorescence staining of calreticulin (CRT, red) and nuclei (DAPI, blue) in tumor sections from mice intravenously administered PBS or SGPF with or without 1064 nm irradiation.


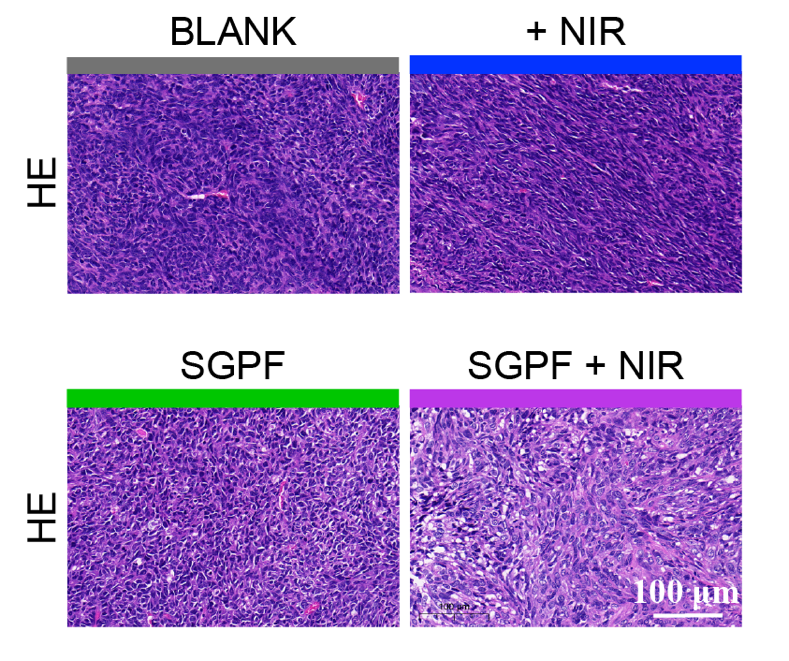


**Figure S20.** Hematoxylin and eosin (H&E) staining of primary tumors following indicated treatments.


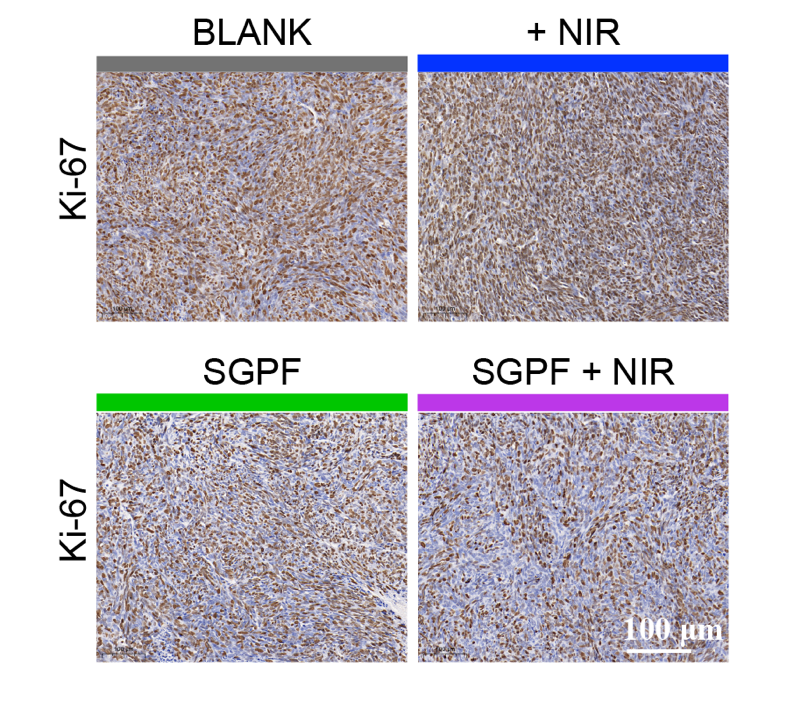


**Figure S21**. TUNEL staining of the distant tumors following different treatment approaches at the end of the study.


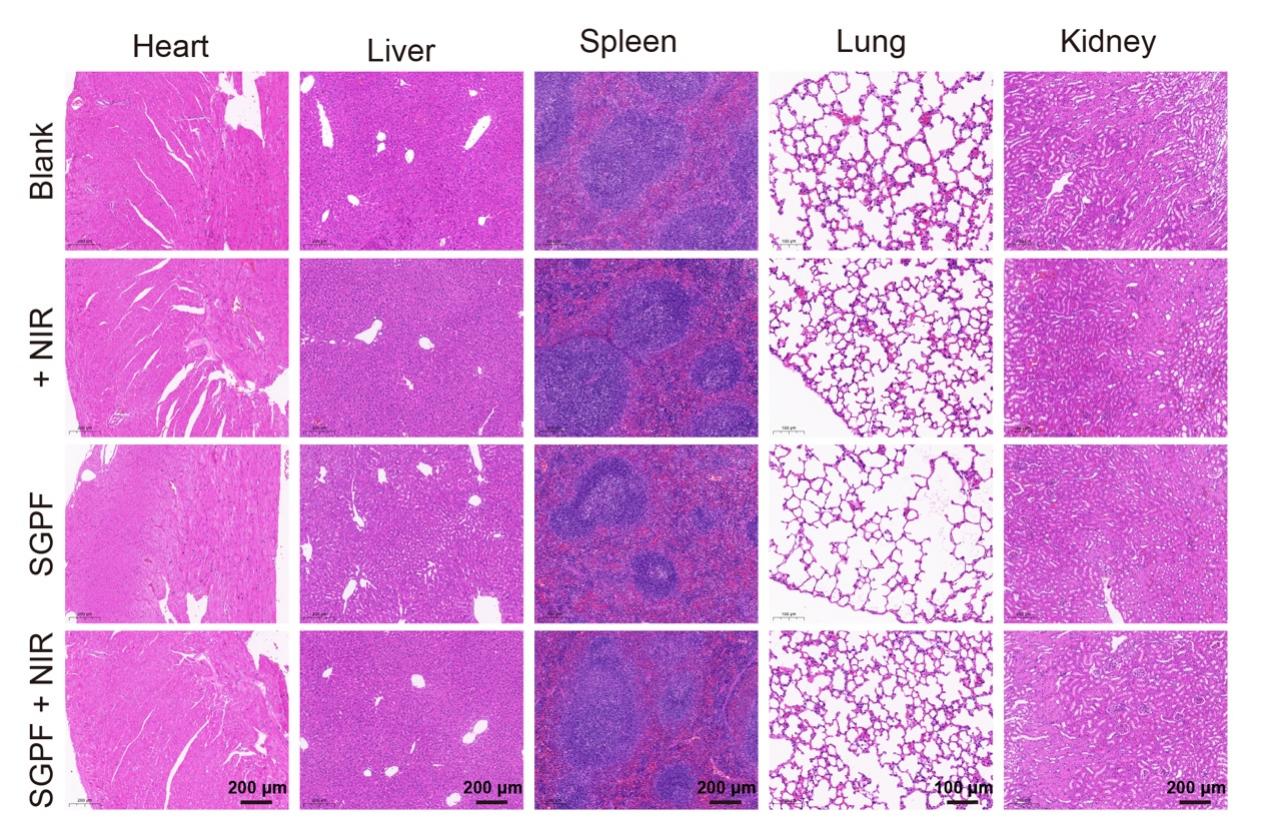


**Figure S22.** *In vivo* biosafety assessment: H&E-stained sections of heart, liver, spleen, lungs, and kidneys following different treatment approaches.


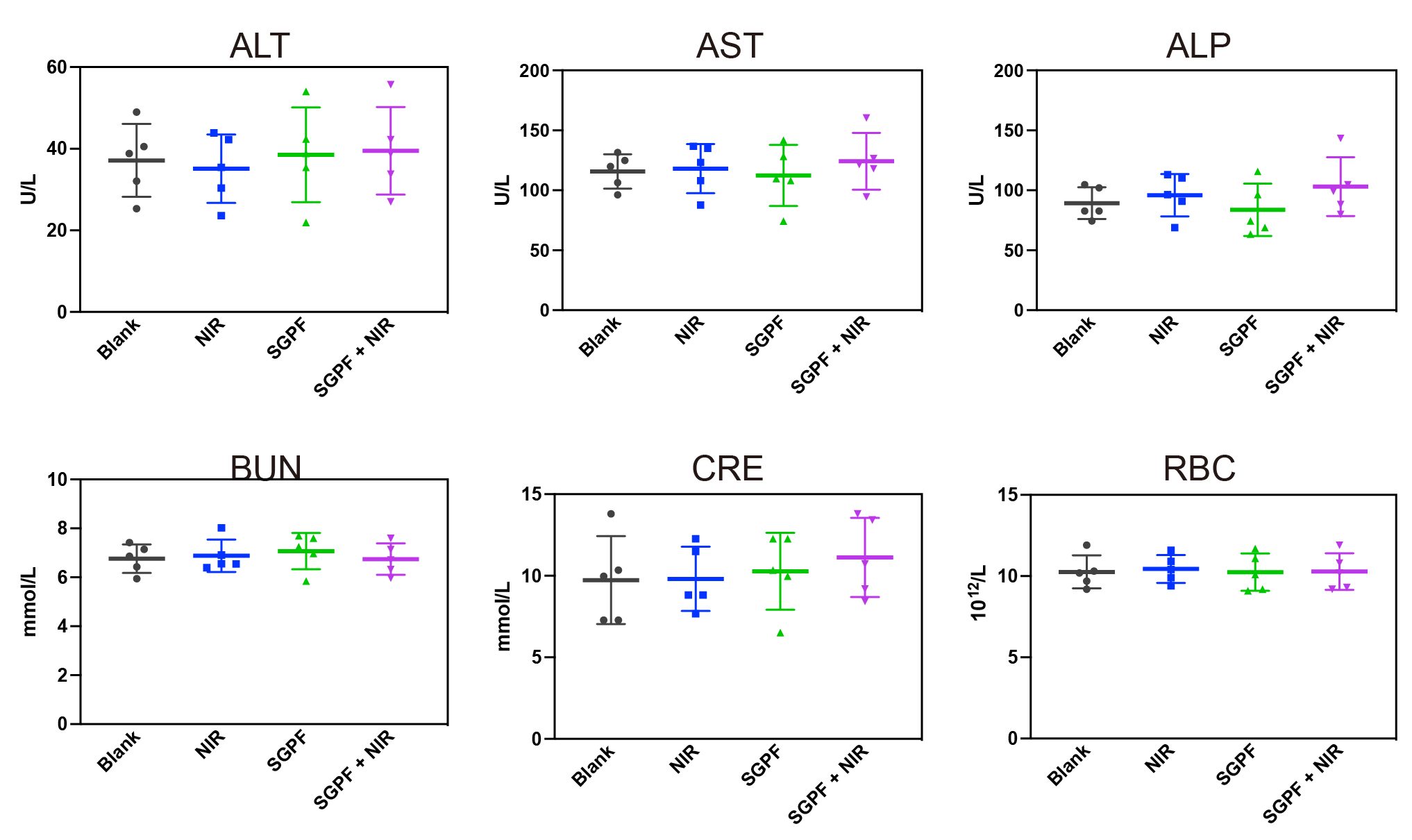


**Figure S23.** Complete blood count and biochemical profiling 72 h post-treatment with PBS, NIR alone, SGPF alone, or SGPF plus NIR.


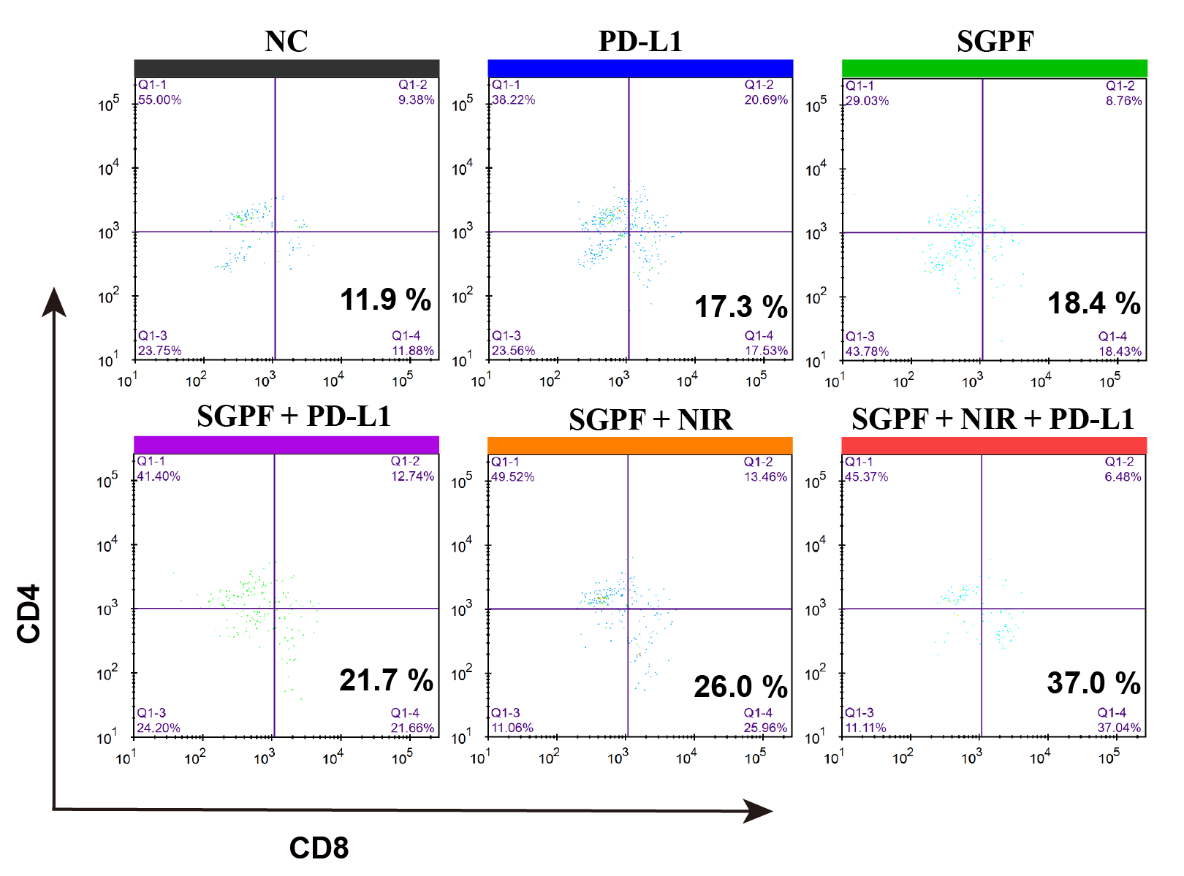


**Figure S24.** Representative flow cytometry plots of cytotoxic T lymphocytes (CTLs; CD3⁺CD8⁺) and T helper cells (Th; CD3⁺CD4⁺) in tumor-infiltrating lymphocytes.


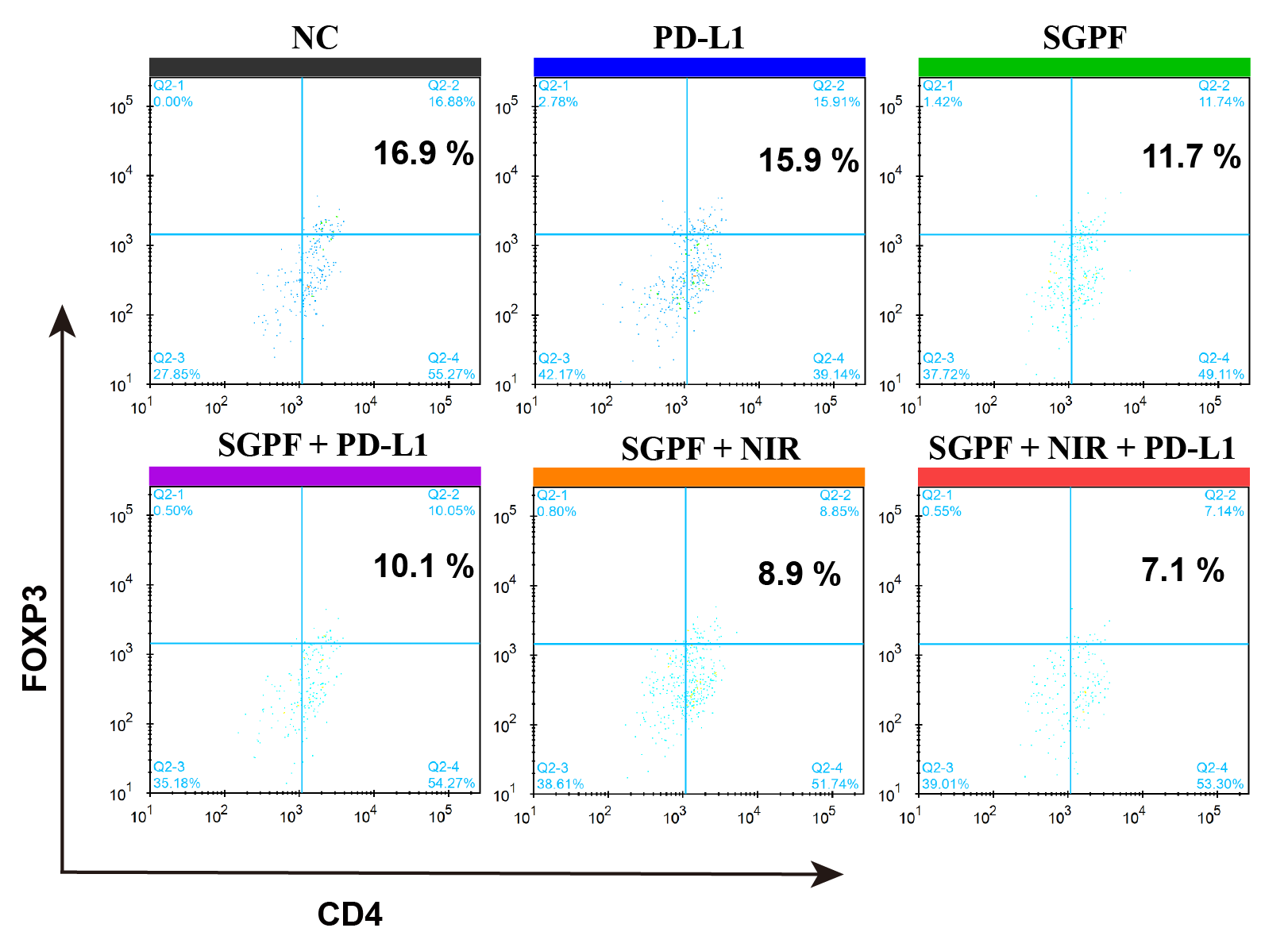


**Figure S25.** Representative flow cytometry analysis of regulatory T cells (Tregs; CD3⁺CD4⁺FOXP3⁺) within tumor microenvironments.


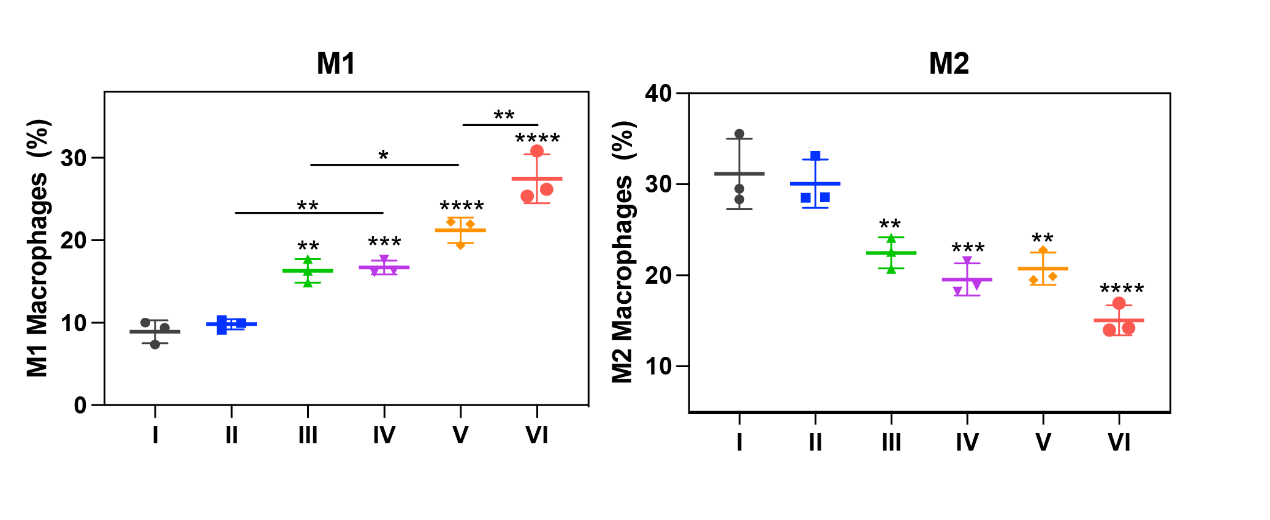


**Figure S26.** Relative frequencies of M1-polarized (CD86⁺CD206⁻) and M2-polarized (CD86⁻CD206⁺) macrophages across experimental groups.
